# Which Exercise Prescriptions Improve Physical Fitness in Patients with Breast Cancer Before, During, and Following Treatment? A Systematic Review and Meta-analysis of Randomized Controlled Trials

**DOI:** 10.1007/s40279-025-02390-4

**Published:** 2026-03-25

**Authors:** Vinicius S. S. Cassaroti, Maurício R. Lopes, Guilherme H. D. Palma, Paola S. Cella, Loriane R. L. C. Godinho, Tatiana G. Azevedo, Jonathan H. C. Nunes, Mayra B. S. Borges, Rafael Deminice

**Affiliations:** 1 Department of Physical Education, Faculty of Physical Education and Sport, State University of Londrina, Rodovia Celso Garcia Cid, Pr 445 km 380, Campus Universitário, Londrina, PR 86057-970 Brazil; 2 State University of Northern Paraná, Jacarzinho, Paraná 86400-000 Brazil; 3 State University of Maringa, Maringá, Paraná 87020-900 Brazil

## Abstract

**Background:**

Exercise is now recognized as an essential component of the cancer care continuum. However, the optimal exercise prescriptions to improve physical fitness in breast cancer remain under debate.

**Objectives:**

In a systematic review and meta-analysis of randomized controlled trials (RCTs), we investigated how exercise affects muscular strength, fat-free mass, functional exercise capacity (FEC) and cardiorespiratory fitness during pretreatment, active treatment, and post-treatment for breast cancer. We also investigated how exercise mode, frequency, intensity, supervision, and duration of intervention affect breast cancer survivors’ physical fitness.

**Methods:**

We searched four electronic databases to identify RCTs examining exercise’s effects on muscular strength, fat-free mass, functional exercise capacity, FEC, and cardiorespiratory fitness in women with breast cancer. We calculated pooled effects (SMD) using the software Comprehensive Meta-Analysis. Subgroup analyses were conducted based on phases of cancer care and exercise mode, frequency, intensity, supervision, and duration of intervention.

**Results:**

Across 68 randomized controlled trials (*N* = 4,158), exercise significantly increased muscular strength (Hedges’ g = 0.66, 95% CI 0.46–0.85), FEC (Hedges’ g = 1.04, 95% CI 0.56–1.52), cardiorespiratory fitness (Hedges’ g = 0.96, 95% CI 0.45–1.47), and fat-free mass (Hedges’ g = 0.12, 95% CI 0.03–0.20). However, these effects varied according to the phase of cancer care and exercise prescription characteristics, including modality, frequency, supervision, intensity, and duration. During active treatment, combined exercise significantly improved muscular strength, FEC, and cardiorespiratory fitness, but not fat-free mass. In contrast, post-treatment exercise significantly enhanced muscular strength, fat-free mass, and FEC, with no significant effect on cardiorespiratory fitness. Data for evaluating exercise’s pretreatment effects were insufficient. The greatest improvements across outcomes were observed with supervised combined aerobic-resistance exercise programs performed at least three times per week for a minimum of 12 weeks.

**Conclusions:**

Although exercise improves breast cancer survivors’ physical fitness, its effects differ depending on its mode, frequency, intensity, supervision, and total duration in addition to the phase of cancer care.

**Supplementary Information:**

The online version contains supplementary material available at 10.1007/s40279-025-02390-4.

## Key Points

**Table:** 

The effects of exercise training on muscular strength, fat-free mass, functional exercise capacity, and cardiorespiratory fitness differ depending on the specific phase of breast cancer care.
Exercise during active treatment significantly increases muscular strength, functional exercise capacity, and cardiorespiratory fitness but not fat-free mass. When exercise is performed post-treatment, it significantly enhances muscular strength, fat-free mass, and functional exercise capacity but not cardiorespiratory fitness.
To achieve optimal improvements in physical fitness among breast cancer survivors, an exercise training intervention should include supervised and combined aerobic-resistance exercises of moderate-to-high intensity performed for at least 12 weeks, with a frequency of at least three sessions per week.

## Introduction

Exercise is a key strategy for promoting health among survivors of breast cancer. In the past decade, medical and scientific organizations worldwide have consistently shown that exercise not only plays a key role in preventing and controlling breast cancer’s recurrence and mortality but also alleviates cancer treatment’s primary adverse effects [1–6]. Nevertheless, recommendations for exercise for cancer survivors remain unspecific and imprecise [7]. Indeed, most exercise oncology guidelines do not address key components of exercise prescription, such as mode, frequency, intensity, supervision, and intervention duration. Consideration of the most recently published guidelines [1–6] shows that only one provides specific recommendations regarding frequency, intensity, and volume [3]. Furthermore, the American Society of Clinical Oncology, in its guidelines for exercise, states that the available evidence does not offer enough specific information to formulate precise exercise prescriptions for patients undergoing active cancer treatment [2]. Added to generic and imprecise recommendations, many trials in exercise oncology have neither applied nor reported key principles of exercise training. In a systematic review, Bland et al. [7] evaluated the inclusion of such principles in breast cancer clinical trials and found that no studies had fully accounted for all principles of exercise training or reported all components of the prescribed exercises in their methods. However, such information is relevant because the intensity, frequency, mode, and overall duration of exercise determine the principle of specificity, which ensures precise physiological adaptations to achieve the desired muscle phenotype and fitness outcomes [8, 9]. For this reason, incorporating established exercise principles into future studies and clinical services in exercise oncology will enhance the precision and effectiveness of prescribed interventions.

Beyond generic prescriptions, responses in cancer survivors vary depending on the phase of their cancer care. Cancer survivorship can be divided into four distinct phases: after diagnosis and before surgery (i.e., prehabilitation), after surgery and before adjuvant treatment, during non-surgical therapy (i.e., adjuvant and neoadjuvant), and during extended survival, which includes transitional survivorship [10]. However, guidelines and meta-analyses tend to aggregate phases of cancer care into general categories and rarely investigate the effects of exercise programs with different frequencies, intensities, modes, and overall durations of exercise during different phases of cancer care [7, 11]. Such vagueness contributes to generic recommendations and imprecise exercise prescriptions and may also mask the full therapeutic potential of exercise as a supportive care intervention in cancer treatment.

Considering all the above, in a systematic review and meta-analysis, we strove to examine how key components of exercise training—mode, frequency, intensity, supervision, and duration of intervention—impact components of physical fitness (muscular strength, fat-free mass, functional exercise capacity [FEC], and cardiorespiratory fitness) among breast cancer survivors. We also investigated the effects of exercise training on physical fitness at different phases of cancer care for breast cancer: pre-treatment, during active treatment, and post-treatment. Overall, we hypothesized that exercise training’s effects on muscular strength, fat-free mass, FEC, and cardiorespiratory fitness differ depending on exercise mode, frequency, intensity, supervision, and duration of intervention and the different phases of breast cancer care. We expect that our results can help to refine prescriptions for exercise for breast cancer survivors, particularly by sharpening their focus on optimizing physical fitness.

## Methods

Our study followed the methodology for systematic reviews proposed by the Cochrane Handbook [12], and in this article, we report our findings following the Preferred Reporting Items for Systematic Reviews and Meta-Analyses (PRISMA) 2020 statement [13] and the recently published Prisma in Exercise, Rehabilitation, Sport Medicine and Sports Science (i.e., PERSiST) guidelines [14]; the PRISMA checklist is presented in the Electronic Supplementary Material (ESM). The protocol for our systematic review was registered in the International Prospective Register of Systematic Reviews (PROSPERO, No. CRD42023411705).

### Sources and Search Strategy

A primary search was conducted in the databases PubMed, SPORTDiscus, Google Scholar, and LILACS to collect bibliographic material relevant to our study’s purposes. Next, a comprehensive search in each database was conducted for publications reporting randomized controlled trials (RCTs) published from 1989, when the first RCT involving patients with cancer was published, through 31 December, 2024. Targeting only publications available in English, Spanish, and Portuguese, the search was performed using combinations of MeSH terms and free-text words for “breast,” “cancer,” “physical activity,” and “exercise.” Appropriate Boolean operators and database filters were applied to optimize the search, as detailed in the ESM. Our strategies were constructed by library services at the State University of Londrina and peer reviewed by one of the authors (i.e., RD) before execution. The reference lists of relevant published reviews and all publications included in our review were also examined for additional references.

### Eligibility Criteria and Outcomes

The inclusion criteria that we adopted follow the PICO approach, which considers the population, intervention, comparison, and outcome targeted for investigation [15]. Regarding the population, all RCTs involving adult women (i.e., aged ≥ 18 years) in any phase of primary oncology treatment for breast cancer (e.g., surgery, infusion therapy, and/or radiotherapy) were eligible. If the RCT involved other types of cancer, it was considered to be eligible provided the breast cancer data were reported separately. Regarding the intervention, all RCTs entailing exercise or physical activity were eligible, with all forms of intervention, counseling, or strategies aimed at improving body movement, maintaining physical capacity or performance, and health accepted. Randomized controlled trials were eligible regardless of the prescribed exercise’s mode, intensity, frequency, supervision, and duration of intervention, in addition to the phase of cancer care (i.e., pre-treatment, during treatment, or post-treatment). Randomized controlled trials that included additional interventions not related to the primary oncology treatment, such as dietary interventions, counseling, massage, acupuncture, or other practices, alongside exercise, and for which the effects could not be isolated, were excluded. Randomized controlled trials involving Asian practices (e.g., yoga and tai chi) and physiotherapy interventions aimed at rehabilitating a specific body region (e.g., spine, arms, or legs) were also excluded. Regarding the comparison, to be eligible, RCTs had to include a control group of individuals who received standard care, were inactive, and/or were less active than the intervention group.

Lastly, the primary outcomes for our study were muscular strength, fat-free mass, FEC, and cardiorespiratory fitness. Muscular strength was represented by the results of manual dynamometry, weightlifting, and one-to-multiple maximum repetition tests for upper and/or lower limb muscle groups. By contrast, fat-free mass was represented by measurements using anthropometry, bioelectrical impedance analysis, or any imaging method (e.g., tomography, dual-energy X-ray absorptiometry, scanners, and nuclear resonance imagery). Functional exercise capacity was represented by the distance reached in the 6- or 12-min walk test, respectively, while cardiorespiratory fitness was represented by the maximum or peak oxygen consumption measured by using oxygen exchange equipment or estimated using a field test (i.e., 1-mile or 400-m walking test). To be included, the publications needed to report at least one of the primary outcomes considered in our study. The secondary outcomes were variables related to prescribed exercise, including mode (i.e., aerobic, resistance, or combined), frequency, intensity, supervision, and duration of intervention.

### Publication Selection and Data Extraction

The process for including publications in our review spanned four stages. First, duplicate publication review papers were excluded using Rayyan (https://www.rayyan.ai/). Second, at least two authors in pairs (i.e., VS and ML, GD and TA, PC and GP, and LG and MB) independently assessed the publications’ eligibility for inclusion based on their titles and abstracts while excluding posters, conference abstracts, non-peer-reviewed journal articles, study protocols, and any publication without full text available. Third, publications that met the specified criteria were thoroughly and independently reviewed by at least two authors in the same pairs and reassessed for inclusion in our review. Any discrepancies that emerged within any pair of authors were resolved by a third author (i.e., RD).

Once publications were confirmed for inclusion, the following data were extracted: the publication’s characteristics (i.e., authors, year, journal of publication, and study design), participants’ characteristics (i.e., sample size, mean age, stage of cancer, and phase of treatment), exercise-related variables (i.e., mode, intensity, frequency, supervision, and duration of intervention), characteristics of the control condition, and outcome measures (i.e., muscular strength, fat-free mass, FEC, and cardiorespiratory fitness). Data about the characteristics of the eligible publications were also extracted independently by pairs of authors (i.e., GD and TA, PC and GP, and LG and MB).

### Assessment and Investigation of Heterogeneity

Statistical heterogeneity among the included RCTs was quantified by prediction intervals when there were more than ten RCTs per analysis but by the *I*^2^ statistic and *Q* test (*χ*^2^) with a significance level of *p* ≤ 0.05 when fewer than ten RCTs were analyzed [16]. *I*^2^ values of < 30% indicated that heterogeneity may not be important, values of 30–60% indicated moderate heterogeneity, and values of > 60% indicated substantial heterogeneity.

### Risk of Bias Assessment

Risk of bias was assessed independently by two reviewers (i.e., VSS and ML) using RoB 2, i.e., the Cochrane Collaboration’s tool for assessing risk of bias, Version 2.12 items representing blinding were excluded because blinding participants and personnel for exercise interventions is not typically feasible [14]. Both reviewers compared their quality assessments, and disagreements were resolved by discussion. If no consensus could be reached, then a third author (i.e., RD) was consulted for a final assessment.

### Statistical Analysis

Effect sizes for all individual studies were calculated by subtracting the control group’s average post-intervention score from the intervention group’s average post-intervention score and dividing the result by the pooled standard deviation of both groups. All effect sizes were pooled using Hedges’ *g*. Following Cohen’s convention, we interpreted effect sizes of 0.2 as being small, 0.5 as being moderate, and 0.8 as being large [17]. When a publication reported only the standard error, the standard deviation was calculated by multiplying the standard error by the square root of the sample size. Because the samples and interventions were assumed to be heterogeneous, effect sizes were pooled with a random-effects model that accounted for any systematic difference in the effects between the studies. Meta-analytical statistics were calculated in the software Comprehensive Meta-Analysis, Version 4 (Biostat, Englewood, NJ, USA).

Next, we calculated the differences in effects between subgroups defined by the phase of cancer treatment (pre-treatment, active treatment, and post-treatment), according to An et al.’s [10] physical activity and cancer control framework. Subgroup analyses were also conducted to evaluate the effects of each moderator of physical exercise. First, for the mode of exercise, aerobic exercise programs had to involve continuous or mostly continuous exercise (e.g., walking, running, and biking). Resistance exercises, by contrast, encompassed weightlifting, CrossFit, and Pilates. Any combination of aerobic and resistance exercise was considered to be combined. Any publications proposing physical therapy with rehabilitation were excluded. Second, exercise was classified as supervised when at least 75% of the sessions were conducted face-to-face (either in person or online) under professional supervision; interventions not meeting this criterion were considered unsupervised. Third, the duration of intervention was categorized as either < 12 or > 12 weeks. Fourth, frequency was either at least two times weekly or at least three times weekly.

Fifth and finally, intensity was categorized depending on the mode of exercise. First, for aerobic exercise interventions, low-to-moderate intensity was recorded when the maximum heart rate (HRmax) was < 70%, the HRmax adjusted for age was < 55%; the heart rate was < 60% when the 6-min walk test was used; or the intensity was set as < 60% of maximum oxygen consumption or VO2peak; rating of perceived exertion [RPE] (6–20 scale) was < 12; the RPE (0–10 scale) was < 4, or total work was ≤ 6 metabolic equivalents of task. Self-guided aerobic exercise programs using guidelines or mobile apps that did not specify the intensity or labeled it as “moderate” were classified as having low-to-moderate intensity as well. By contrast, moderate-to-high intensity was recorded when HRmax was > 70%, the HRmax adjusted for age was > 55%; the heart rate was > 60% when the 6-min walk test was used; or the intensity was set as > 60% of maximum oxygen consumption or peak oxygen consumption; RPE (6–20 scale) was > 12; the RPE (0–10 scale) was > 4, or total work was ≥ 6 metabolic equivalents of task. Second, for resistance exercise programs, the intensity was considered to be low to moderate when it was performed at < 70% of the one repetition maximum; or the RPE scale (6–20 scale) was < 14; or the RPE scale (0–10 scale) was < 5, and moderate to high when exercise was prescribed with >> 70% of one repetition maximum; > 14 RPE (6–20 scale); > 5 RPE (0–10 scale). Elastic bands or bodyweight-based exercises without any additional information about intensity were considered to be low-to-moderate intensity. Third, for combined exercise protocols, exercise was considered to be moderate to high when one or both of the exercise modes (i.e., aerobic or resistance) were classified as having moderate-to-high intensity. When both modes were classified as having low-to-moderate intensity, the combined exercise program’s intensity was considered to be low to moderate. For all analyses, the significance level was set at *p* < 0.05.

## Results

### Publication Inclusion

**Fig. 1:**
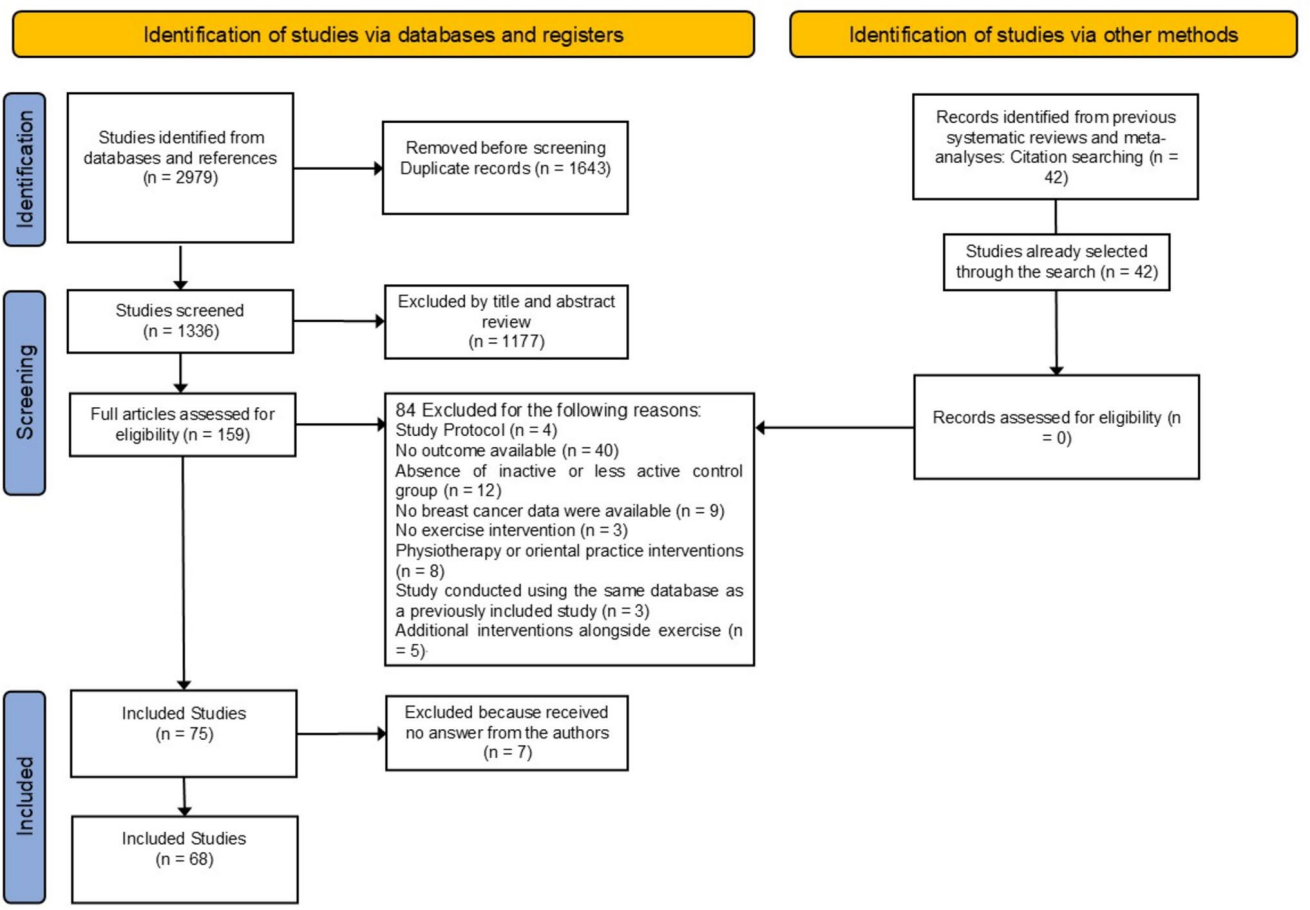
Preferred Reporting Items for Systematic Reviews and Meta-Analyses (PRISMA) flow chart of the inclusion of publications

Our initial search of the databases returned 2979 publications. After removing duplicates, we selected 159 publications based on their titles and abstracts and thoroughly evaluated them. We also identified 42 RCTs cited in previous systematic reviews and meta-analyses; all of these had already been captured in our search. Of the 159 studies screened, 91 were excluded for various reasons (see ESM), whereas the remaining 68 were retained. Figure 1 provides a flowchart of how publications were chosen for our review.

The principal investigator or corresponding author of eight RCTs was contacted because of incomplete data. After a personal communication, we obtained additional information for only one RCT, which led to the exclusion of seven other publications. Thus, our meta-analysis ultimately included 68 RCTs [18–85] with 108 evaluation arms, 39 of which examined the effects of exercise on muscular strength [18–20, 24–29, 33, 37, 39, 43, 48–51, 54, 55, 57, 59–65, 67, 69–71, 73, 77, 79–83, 85], 39 on fat-free mass [18, 19, 21–23, 30–32, 34–38, 40, 41, 45–47, 50–53, 55, 57, 60, 61, 64, 68–72, 76–79, 81, 82], 12 on FEC [18, 25, 27, 42, 44, 48, 56, 58, 73, 81, 83, 84], and 18 on cardiorespiratory fitness [27, 28, 31, 33, 41, 46, 51, 54, 57, 59, 61, 65, 66, 68, 69, 74, 75, 79].

### Characteristics and Populations of RCTs

The 68 RCTs included in our meta-analysis were published between 2001 and 2024, all in English, and included 4158 women with breast cancer: 2058 in control groups and 2100 in exercise intervention groups. The average age of participants in the control and intervention groups was 52.3 ± 8.4 and 52.4 ± 8.1 years, respectively. All RCTs included patients with stage I–III breast cancer; most (64%) assessed the effects of exercise after cancer treatment [21–23, 26, 29, 34–42, 44–50, 52, 53, 58–61, 63–70, 72, 74–78, 84, 85] whereas 33% evaluated its effects during active treatment (e.g., chemotherapy and/or radiotherapy) [18, 19, 24, 25, 27, 28, 30, 32, 33, 43, 51, 54–57, 62, 71, 73, 79–82] and only two evaluated its effects during pre-treatment [31, 83].

Regarding the exercise protocols, the average duration of the interventions was 18.6 weeks, with a range of 1 week [43] to 52 weeks [21, 37, 63, 77]. Most RCTs (58%) required exercise at a frequency of three or more times weekly [18, 20, 21, 26, 28, 30, 32, 34, 35, 37, 39, 41, 43–52, 55, 56, 58, 59, 62, 63, 65, 66, 69, 73–77, 79, 84] and, by mode, 48% involved combined aerobic-resistance exercise [18, 19, 21, 23, 26, 27, 29, 30, 33–36, 38, 40–42, 44, 45, 49, 50, 53, 57, 61, 62, 64, 76–81, 83], whereas 28% involved aerobic exercise [22, 31, 32, 46, 47, 51, 52, 56, 58, 63, 65–68, 71, 73–75, 84]. Regarding supervision, 67% involved supervised exercise [19–29, 31, 33–37, 39–41, 43, 46–48, 50, 54, 57, 58, 60, 61, 64, 67, 69–72, 75, 76, 79–83, 85], and with respect to intensity, more than half (60%) described protocols with moderate-to-high-intensity workouts [23–27, 29, 30, 32–35, 39–42, 45, 46, 48, 50–54, 56–58, 60, 64, 68–71, 73, 75, 79, 80, 82]. The characteristics of the studies are presented in the ESM.

### Risk of Bias in the Publications

**Fig. 2:**
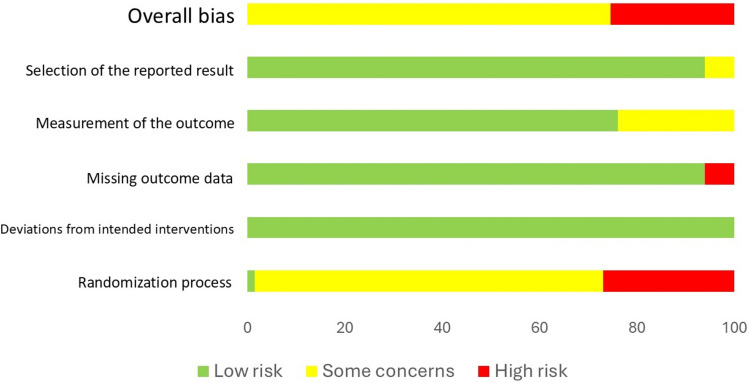
Risk of bias assessment of included studies using Version 2 of the Cochrane risk of bias tool

Most RCTs (75%) were classified as having “some concerns” regarding risk of bias. Twenty-five percent of the studies were judged as having a “high” risk of bias, and none had a “low” risk of bias. Most RCTs (75%) were missing some information on the randomization process and were considered as having “some concerns”. The risk of bias is summarized in Fig. 2, and the risk of bias for each RCT is shown in the ESM.

### Characteristics of Arms of the RCTs

The four physical capacities evaluated—muscular strength, fat-free mass, FEC, and cardiorespiratory fitness—were analyzed separately in our meta-analysis. First, the 39 RCTs that assessed muscular strength involved 1176 control and 1183 intervention participants aged 51.8 years on average (mean control group age = 51.8 years; mean intervention group age = 51.9 years). Almost half of the RCTs (49%) assessed the effects of exercise following cancer treatment [20, 26, 29, 37, 39, 48–50, 59–61, 63–65, 67, 69, 70, 73, 77, 85] for 18.2 weeks on average, with durations that ranged from 1 to 52 weeks. Regarding frequency, most of the RCTs (53%) examined exercise performed at least three times weekly [18, 20, 26, 28, 37, 39, 43, 48–51, 55, 59, 62, 63, 65, 69, 73, 77, 79]; by mode, 45% involved combined exercise [18, 19, 26, 27, 29, 33, 48, 49, 57, 61, 62, 64, 77, 79–81, 83]; and by supervision, most (81%) were supervised [19, 20, 24–29, 33, 37, 39, 43, 48, 50, 51, 54, 57, 60, 61, 64, 67, 69–71, 77, 79–83, 85].

Second, the 39 RCTs that assessed fat-free mass included 1071 control group participants and 1115 intervention group participants who were 52.8 years of age on average (mean control group age = 52.7 years; mean intervention group age = 52.6 years). Most of the RCTs (72%) examined exercise’s effects following cancer treatment [21–23, 34–38, 40, 41, 45–47, 50, 52, 53, 60, 61, 64, 68–72, 76–78] and the average duration of the interventions was 19.7 weeks, with a range of 4 to 52 weeks. Little more than half of the RCTs (55%) implemented exercise programs with a frequency of two or fewer sessions per week [19, 22, 23, 30, 36, 37, 40, 53, 57, 60, 61, 64, 68, 70–72, 76, 78, 81, 82], 57% used combined exercise [18–21, 23, 31, 34–36, 38, 40, 41, 45, 53, 57, 61, 64, 76–79, 81], and 69% were supervised [19, 21–23, 28, 30, 34–37, 40, 41, 46, 47, 50, 57, 60, 61, 64, 69–72, 76, 79, 81, 82].

Third, the 12 RCTs that examined FEC included 613 breast cancer survivors—305 in control groups and 308 in intervention groups—with an average age of 51.1 years (mean control group age = 50.9 years; mean intervention group age = 51.2 years). All such RCTs investigated cases of breast cancer in stages I–III, with 50% focusing on exercise’s effects during treatment. The interventions lasted 18 weeks on average, with a range of 6 to 27 weeks, and most RCTs employed exercise programs that were unsupervised (58%) and combined aerobic and resistance exercises (58%).

Lastly, the 18 RCTs that examined cardiorespiratory fitness included 1288 breast cancer survivors—632 in their control groups and 656 in their intervention groups—aged 52.1 years on average (mean control group age = 51.9 years; mean intervention group age = 51.9 years). Most such RCTs included cases of stage I–III breast cancer (87%) and assessed exercise’s effects following cancer primary treatment (55%). Most of the interventions occurred three or more times weekly (57%), were supervised (70%), and provided only aerobic exercise (44%). On average, the interventions lasted 11.8 weeks, with a range of 4 to 27 weeks.

### Effects of Exercise Interventions

#### Primary Outcomes

Table 1 displays the results of our meta-analysis for the primary outcomes. Overall, exercise significantly enhanced muscular strength, FEC, and cardiorespiratory fitness in breast cancer survivors with moderate-to-large effects. Low but significant effects favoring exercise also emerged for fat-free mass. However, another sub-analysis revealed that exercise’s impact on primary outcomes varied depending on the phase of cancer care. In particular, exercise significantly increased muscular strength during active treatment and post-treatment with moderate-to-large effects. Moreover, exercise training significantly enhanced fat-free mass post-treatment with a low effect size, albeit not in pre-treatment or active treatment. Furthermore, exercise significantly improved FEC during both active treatment and post-treatment and with large effects. In addition, cardiorespiratory fitness significantly improved with exercise during active treatment and with a large effect, albeit not in pre- or post-treatment. However, data to assess exercise’s effects on muscular strength and FEC during pre-treatment were insufficient (Table 1). Lastly, the predicted interval and *I*^2^ demonstrated potential variability in all analyses and moderate-to-high heterogeneity, except for fat-free mass, which demonstrated low heterogeneity.

**Table 1:** Pooled effects of exercise on strength, fat-free mass, and functional exercise capacity in patients with breast cancer pre, during, and post-treatment phases

	*N*	Hedges’ g (95% CI)	Predicted interval	*I*^2^	Between-group difference (*p*-value)
**Strength**					0.25
Pre-treatment	1	0.32 (− 0.17, 0.81)	0	0	
During	19	0.77 (0.47, 1.08)	− 0.53, 2.08	85.6	
Post-treatment	19	0.54 (0.31, 0.77)	− 0.26, 1.35	57.7	
Overall	39	0.66 (0.46, 0.85)	− 0.41, 1.73	78.0	
**Fat-free mass**					0.05
Pre-treatment	1	− 0.05 (− 0.75, 0.63)	0	0	
During	11	− 0.02 (− 0.17, 0.12)	0	0	
Post-treatment	27	0.19 (0.08, 0.30)	0.11, 0.38	5.8	
Overall	39	0.12 (0.03, 0.20)	0.01, 0.22	1.2	
**Functional exercise capacity**					0.00
Pre-treatment	1	3.66 (2.86, 4.46)	0	0	
During	6	0.81 (0.47, 1.14)	− 0.09, 1.71	45.3	
Post-treatment	5	0.83 (0.19, 1.46)	− 1.47, 3.13	82.9	
Overall	12	1.04 (0.56, 1.52)	− 0.79, 2.87	86.9	
**Cardiorespiratory fitness**					
Pre-treatment	1	0.09 (− 0.60, 0.79)	0	0	0.01
During	7	1.94 (0.84, 3.04)	− 2.03, 5.91	97.5	
Post-treatment	10	0.31 (− 0.01, 0.62)	− 0.58, 1.19	52.9	
Overall	18	0.96 (0.45, 1.47)	− 1.30, 3.22	93.6	

Between-group differences were assessed using a mixed-effects analysis, with *p* < 0.05 considered statistically significant

*CI* confidence interval

#### Secondary Outcomes

The results of sub-analyses examining the effects of exercise mode, frequency, intensity, supervision, and duration of intervention on muscular strength and free-fat mass are shown in Table 2. Although all modes of exercise promoted significant gains in strength, resistance exercise’s effects on strength were significantly greater (*p* < 0.01) than the effects of aerobic and combined exercise. Furthermore, supervised exercise significantly improved strength and with a large effect, while unsupervised exercise had no effect. The effects of exercise on muscular strength also did not differ significantly by duration of intervention, exercise frequency, or exercise intensity (Table 2). Table 2 additionally shows that combined exercise significantly increased fat-free mass with a low effect, whereas resistance and aerobic exercise did not. Similar to muscular strength, supervised exercise significantly improved fat-free mass, while unsupervised exercise did not. Significant improvements in fat-free mass were observed only when the intervention exceeded 12 weeks and the frequency was three or more times weekly, both with low effect sizes. No significant effects of exercise intensity on fat-free mass emerged (Table 2).

**Table 2:** Pooled effects of exercise mode, level of supervision, duration, intervention duration, frequency, and intensity on breast cancer survivors’ muscle strength and fat-free mass

	*N*	Hedges’ g (95% CI)	Predicted interval	*I*^2^	Between-group difference (*p*-value)	
Strength						
**Exercise mode**					0.00	
Aerobic	6	1.11 (0.32, 1.89)	− 1.65, 3.88	92.4		
Resistance	16	0.88 (0.66, 1.10)	0.15, 1.60	555.0		
Combined	17	0.35 (0.11, 0.60)	− 0.54, 1.25	68.1		
**Delivery mode**					0.31	
Supervised	31	0.71 (0.49, 0.93)	− 0.38, 1.80	79.8		
Non-supervised	8	0.45 (− 0.02, 0.90)	− 1.03, 1.93	75.8		
**Intervention duration (weeks)**					0.61	
< 12	21	0.71 (0.42, 0.99)	− 0.52, 1.94	82.1		
> 12	18	0.61 (0.36, 0.86)	− 0.38, 1.60	72.1		
**Frequency (times/week)**					0.63	
≤ 2	19	0.61 (0.37, 0.86)	− 0.33, 1.58	72.4		
≥ 3	20	0.71 (0.38, 1.03)	− 0.68, 2.10	81.2		
**Intensity**					0.93	
Low to moderate	15	0.65 (0.39, 0.91)	− 0.30 to 1.60	73.0		
Moderate to high	24	0.67 (0.39, 0.95)	− 0.59 to 1.93	81.0		

Fat-free mass						
**Exercise mode**					0.38	
Aerobic	9	0.11 (− 0.10, 0.32)	0	0		
Resistance	9	− 0.01 (− 0.18, 0.17)	0	0		
Combined	21	0.15 (0.02, 0.28)	− 0.21, 0.51	28.6		
**Delivery mode**					0.55	
Supervised	27	0.13 (0.02, 0.25)	− 0.09, 0.37	10.8		
Non-supervised	12	0.08 (− 0.06, 0.22)	0	0		
**Intervention duration (weeks)**					0.42	
< 12	20	0.06 (− 0.0, 0.20)	0	0		
> 12	19	0.14 (0.01, 0.27)	− 0.24, 0.52	25.7		
**Frequency (times/week)**					0.38	
≤ 2	20	0.08 (− 0.04, 0.20)	0	0		
≥ 3	19	0.13 (0.01, 0.30)	− 0.33, 0.60	38.5		
**Intensity**					0.66	
Low to moderate	14	0.14 (0.08, 0.28)	0	0		
Moderate to high	25	0.11 (0.01, 0.23)	− 0.25, 0.45	24.4		

Between-group difference was tested by a mixed-effect analysis, considering *p* < 0.05

*CI* confidence interval

Stratified analyses also showed no statistically significant effects on FEC between interventions by mode, supervision, duration of intervention, frequency, or intensity (Table 3). With respect to cardiorespiratory fitness, significant improvements were observed after aerobic and combined exercise, but not after resistance exercise, with the largest effects found for aerobic exercise. Significant improvements in cardiorespiratory fitness were also observed with supervised exercise programs compared with unsupervised programs and in exercise programs exceeding 12 weeks compared with programs less than 12 weeks long. No significant effects of exercise frequency or intensity on cardiorespiratory fitness were found either. Again, the predicted interval and *I*^2^ values indicated potential variability in most of the analyses, with moderate-to-high levels of heterogeneity, whereas fat-free mass demonstrated low heterogeneity.

**Table 3:** Pooled effects of exercise mode, level of supervision, duration, intervention duration, frequency, and intensity on breast cancer survivors’ functional exercise capacity and cardiorespiratory fitness

	*N*	Hedges’ g (95% CI)	Predicted interval	*I*^2^	Between-group difference (*p*-value)
Functional exercise capacity					
**Exercise mode**					0.14
Aerobic	4	0.61 (0.34, 0.89)	0	0	
Resistance	1	1.08 (0.52, 1.65)	0	0	
Combined	7	1.31 (0.44, 2.18)	− 1.78, 4.41	92.2	
**Delivery mode**					0.22
Supervised	5	1.40 (0.48, 2.32)	− 2.09, 4.90	91.5	
Non-supervised	7	0.75 (0.27, 1.24)	− 0.83, 2.35	77.2	
**Intervention duration (weeks)**					0.81
< 12	4	1.13 (0.26, 2.08)	− 2.87, 5.14	86.3	
> 12	8	1.05 (0.38, 1.62)	− 1.19, 3.20	88.4	
**Frequency (times/week)**					0.01
≤ 2	5	1.87 (0.81, 2.92)	− 2.16, 5.90	91.7	
≥ 3	7	0.49 (0.28, 0.70)	0	0	
**Intensity**					0.97
Low to moderate	6	1.04 (0.19, 1.88)	− 2.02, 4.10	92.1	
Moderate to high	6	1.02 (0.50, 1.54)	− 0.66, 2.72	75.3	

Cardiorespiratory fitness					
**Exercise mode**					0.45
Aerobic	8	0.69 (0.02, 1.36)	− 1.62, 3.01	87.9	
Resistance	4	2.15 (− 0.66, 4.96)	− 11.1, 15.8	98.4	
Combined	6	0.47 (0.11, 0.84)	− 0.61, 1.58	66.5	
**Delivery mode**					0.72
Supervised	12	1.03 (0.33, 1.72)	− 1.68, 3.74	95.0	
Non-supervised	6	0.83 (− 0.02, 1.68)	− 2.16, 3.82	90.3	
**Intervention duration (weeks)**					0.32
< 12	15	0.64 (0.28, 1.01)	− 0.75, 2.03	98.9	
> 12	3	2.21 (− 0.86, 5.28)	− 37.4, 41.9	81.7	
**Frequency (times/week)**					0.75
≤ 2	7	1.06 (0.01, 2.11)	− 2.76, 4.89	96.8	
≥ 3	11	0.86 (0.32, 1.40)	− 1.08, 2.81	87.9	
**Intensity**					0.01
Low to moderate	6	0.26 (0.08, 0.44)	0	0	
Moderate to high	12	1.37 (0.53, 2.20)	− 1.95, 4.69	95.1	

Between-group difference was tested by a mixed-effect analysis, considering *p* < 0.05

## Discussion

In our systematic review and meta-analysis, we examined how some key components of exercise training, including the exercise’s mode, frequency, intensity, supervision, and duration of intervention, impact the physical fitness of breast cancer survivors. We also investigated exercise training’s effects on those outcomes in different phases of cancer care. Our results show that exercise training’s effects on muscular strength, fat-free mass, FEC, and cardiorespiratory fitness differ depending on the specific phase of cancer care. In particular, exercise during active treatment significantly increases muscular strength, FEC, and cardiorespiratory fitness but not fat-free mass. When exercise is performed post-treatment, it significantly enhances muscular strength, fat-free mass, and FEC but not cardiorespiratory fitness. However, data to assess exercise’s effects on muscular strength, fat-free mass, FEC, and cardiorespiratory fitness in the pre-treatment phase were insufficient. Moreover, our sub-analysis revealed that although both aerobic and resistance exercise lead to significantly increased muscular strength and FEC, combined aerobic-resistance exercise is the most effective and significantly enhances all aspects of physical fitness studied. Similarly, supervised exercise led to significant increases in muscular strength, fat-free mass, FEC, and cardiorespiratory fitness, whereas unsupervised exercise did not. Furthermore, benefits in physical fitness were found to be achievable with exercise programs lasting at least 12 weeks, with a frequency of twice a week and low-to-moderate intensity. An exception is improved fat-free mass, which requires a program of moderate-to-high-intensity exercise exceeding 12 weeks at a frequency of three times per week. Although most of those results demonstrated potential variability and moderate heterogeneity, our findings nevertheless have significant implications for prescriptions of exercise training and the design of future exercise programs for breast cancer survivors.

Our results align with the findings of previous systematic reviews that have revealed the beneficial effects of different types of exercise training on breast cancer survivors’ muscular strength [86], fat-free mass [86–88], FEC [82], and cardiorespiratory fitness [90, 91]. Although previous meta-analyses have not studied the effects of exercise during specific phases of cancer treatment, exercise’s phase-specific effects are relevant. Our study revealed that when the phases of cancer care have not been considered, exercise has been found to enhance all fitness capacities evaluated (see Table 1, “Overall effect”). However, considering the phases of cancer care, exercise’s effect on the components of physical fitness varies. As detailed earlier, exercise during active treatment improves muscular strength, FEC, and cardiorespiratory fitness but not fat-free mass. However, post-treatment exercise enhances muscular strength, fat-free mass, and FEC but not cardiorespiratory fitness. Thus, not considering the phases of cancer care is a critical oversight because it may result in inaccurate or overly generalized recommendations for exercise. Indeed, many clinical trials and recent exercise guidelines and recommendations for cancer survivors do not address the different phases of cancer care. For instance, the most recent American College of Sports Medicine’s Exercise Guidelines for Cancer Survivors do not differentiate recommendations based on specific phases of care [1]. Similarly, the exercise guidelines of the American Society of Clinical Oncology provide recommendations for active treatment only, not pre- or post-treatment [2]. On that count, our meta-analysis, at least to our knowledge, is the first to consider the different phases of cancer care when investigating the effects of exercise.

Our results demonstrate that interventions with supervised exercises improved all outcomes studied, whereas interventions with unsupervised exercise significantly improved FEC only. Those findings align with the results of previous meta-analyses [92] showing that, for oncological patients, supervised exercise training yields more favorable results than self-guided or unsupervised training. In particular, supervised exercise is recommended by both reviews and guidelines for exercise oncology [1, 2], especially for improving outcomes such as quality of life and cancer-related fatigue [93], possibly because patients with cancer engaging in supervised (vs unsupervised) exercise show higher adherence, motivation, and engagement in their exercise routines [93]. According to Sweegers et al. [94], supervised (vs unsupervised) programs offer more rigorous exercise prescriptions, access to superior equipment, real-time feedback, and opportunities for social interaction, all of which may enhance the effectiveness of supervised over unsupervised exercise. Despite supervised exercise’s clear advantages, supervision should not be required if it prevents participation in exercise programs because of time constraints, elevated costs, psychological factors, and/or other reasons that can make supervised interventions inaccessible [95, 96]. Therefore, though supervised exercise should be recommended, it is equally important to promote and support unsupervised exercise for cancer survivors when supervision is impossible.

Our sub-analysis comparing the effects of different modes of exercise on the physical fitness of patients with breast cancer yielded nuanced findings. Although resistance training was found to significantly improve muscular strength and FEC and aerobic exercise to enhance cardiorespiratory fitness and FEC, combined exercise was the only mode found to significantly improve all studied outcomes of physical fitness. Moreover, combined exercise was the only mode that significantly enhanced fat-free mass; that finding seems dubious, however, given that combined exercise has also been called “concurrent” exercise in light of evidence that aerobic exercise can attenuate muscle mass and gains in strength [9, 97, 98]. Added to that, the “concurrent” theory has never considered survivors of cancer. Our findings suggest that, for breast cancer survivors, endurance training has no interference effect on resistance training-induced muscle hypertrophy or strength in resistance or aerobic exercise interventions. Furthermore, our meta-analysis included more studies on combined exercise than studies focusing solely on aerobic or resistance training, which resulted in lower heterogeneity. Thus, combined exercise appears to be the best option for cancer survivors, and some guidelines already recommend multi-modal exercise interventions for patients with cancer [3, 6].

Our sub-analysis revealed that muscular strength and FEC can be significantly improved with exercise programs conducted twice weekly for less than 12 weeks. However, to achieve significant gains in fat-free mass, a program needs to have a frequency of three times per week and a duration exceeding 12 weeks. Similarly, although improved cardiorespiratory fitness can occur with exercise performed twice weekly or less, the program also needs to exceed 12 weeks. Interestingly, though longer, higher frequency programs yield better results, studies on healthy individuals have shown that gains in muscle mass can be achieved after only 6 weeks of resistance exercise performed twice weekly [99–102]. Those outcomes suggest that directly translating prescriptions for exercise for the general population into prescriptions for cancer survivors may overlook exercise’s full therapeutic potential as a form of supportive care in oncology. Along with elevated fatigue and other adverse effects of cancer treatments that reduce exercise tolerance, patients with cancer may also experience anabolic resistance, a complex condition characterized by a diminished response to anabolic stimuli, including exercise. Such resistance can result in muscle wasting, strength loss, and impaired cardiopulmonary capacity [103, 104]. Those findings emphasize the importance of tailoring prescriptions for exercise for cancer survivors to prioritize improvements in muscle mass and cardiorespiratory fitness.

The intensity of exercise is critical to driving physiological adaptations and protecting health. Recent studies have shown that intensity-specific physical activity is linked to a reduced risk of mortality from cancer [105]. However, though the intensity of exercise is doubtlessly important, its control and application in exercise oncology remain challenging. Our meta-analysis revealed no significant differences between low-to-moderate-intensity and moderate-to-high-intensity exercise except for gains in fat-free mass. Those non-significant results seem to stem from generic imprecise reporting in the RCTs examined, which has prompted high variability in data about intensity. Many researchers in exercise oncology largely overlook the control and reporting of exercise intensity. During our review, we detected several different methods of controlling exercise intensity; however, only 49% of the publications reviewed provided precise information regarding exercise intensity. The slight majority of publications, by contrast, frequently presented imprecise or ambiguous information, including “moderate intensity” or “8–12 RM with 1–2 min rest between sets” [37, 50], “50 to 75% of maximal heart hate” [18, 34, 35], and statements such as “participants were instructed to perform all exercises until volitional failure” [70]. Such variability prevents any clear conclusion about whether high-intensity exercise is essential to achieving significant improvements in muscular strength, fat-free mass, and cardiorespiratory fitness in breast cancer survivors.

### Limitations

Although we believe that our meta-analysis affords valuable insights into prescribed exercise for individuals with breast cancer, some limitations of our findings warrant mention. First, our primary objective was to assess how components of exercise training impact muscular strength, fat-free mass, FEC, and cardiorespiratory fitness, not whether those changes prompt significant clinical outcomes such as mortality or tumor recurrence. We assumed that improving physical fitness ultimately results in improved health outcomes for breast cancer survivors. Second, how many publications that we reviewed have reported data about training intensity, or the lack of such data, may have influenced the results of our meta-analysis. As mentioned above, only 49% of the publications provided precise data on exercise intensity. The types of aerobic exercises (e.g., walking, cycling, or treadmill use) and the number of muscles targeted in resistance exercises also varied significantly among the RCTs, which raises questions about whether the effects of exercise intensity and volume might differ if they are applied and assessed consistently. Consequently, we acknowledge that the results related to intensity in our meta-analysis should be interpreted with caution. Third, as highlighted in other reviews, reporting on exercise adherence is frequently insufficient or lacking altogether [88]. Approximately one third (34%) of the included publications provided data on adherence to the exercise training program. Adherence to exercise training unquestionably plays a crucial role in the physical fitness of breast cancer survivors. A recent meta-analysis reported an overall pooled exercise adherence of 64% [95% confidence interval (CI) 58, 70] among breast cancer survivors. Adherence was 67% [95% CI 62, 72, *I*^2^ = 95%] for center-based/supervised programs and 60% [95% CI 48, 72, *I*^2^ = 93%] for home-based programs, with no significant differences between adherence during or after primary cancer treatment [106]. Physical fitness, baseline physical activity, and body mass index were identified as key factors associated with adherence in this population, demonstrating their importance in shaping individualized exercise prescriptions to optimize effectiveness.

Fourth and finally, the included RCTs used various methods to measure strength, fat-free mass, FEC, and cardiovascular fitness. For instance, strength was assessed using hand grip strength, with a one-repetition maximum, and isometric force; such variation may have created significant clinical heterogeneity. Sub-analyses of each measurement for further insights are presented in the ESM.

### Recommendations

To achieve optimal improvements in physical fitness among breast cancer survivors, an exercise training intervention should include supervised and combined aerobic-resistance exercises of moderate-to-high intensity performed for at least 12 weeks, with a frequency of at least three sessions per week. Such programs can significantly improve muscular strength and FEC during and after primary treatment, enhance cardiovascular fitness during treatment, and promote minor increases in fat-free mass after treatment. Notably, we found no significant effects of exercise on fat-free mass during primary treatment or on cardiorespiratory fitness after treatment in any sub-analysis. Those findings indicate that exercise interventions have a limited impact on increasing muscle mass in breast cancer survivors. Nevertheless, some enhancement can be achieved with unsupervised low-to-moderate-intensity exercise at a frequency of twice a week, albeit with fewer improvements in physical fitness.

## Conclusions

Exercise improves breast cancer survivors’ muscular strength, fat-free mass, FEC, and cardiorespiratory fitness. However, those effects differ depending on the specific phases of the cancer care and the exercise mode, frequency, supervision, intensity, and total duration. Combined exercise during active treatment significantly increases muscular strength, FEC, and cardiorespiratory fitness but not fat-free mass. When exercise is performed post-treatment, it significantly enhances muscular strength, fat-free mass, and FEC but not cardiorespiratory fitness. Optimal improvements in muscular strength, fat-free mass, FEC, and cardiorespiratory fitness are observed with combined and supervised exercise regimens performed at least three times a week for at least 12 weeks. However, breast cancer survivors may nevertheless experience some improvement or maintenance of those fitness parameters by engaging in unsupervised aerobic and/or resistance exercises at low-to-moderate intensities.

## Supplementary Information

Below is the link to the electronic supplementary material.

Supplementary file1 (PDF 1037 KB)

## Declarations

### Availability of data and material

Data are available in the supplementary material. The raw data can be provided upon reasonable request by contacting the corresponding author.
