# Supplementary material for: Which Exercise Prescriptions Improve Physical Fitness in Patients with Breast Cancer Before, During, and Following Treatment? A Systematic Review and Meta-analysis of Randomized Controlled Trials
Source: Sports Med. 2026 Mar 25;56(5):1175–90. doi: 10.1007/s40279-025-02390-4 (PMC13198464; doi:10.1007/s40279-025-02390-4)
Supplement: Supplementary file 1 — Supplementary file1 (PDF 1037 KB) [file 40279_2025_2390_MOESM1_ESM.pdf]

PRISMA 2020 Checklist

Section and Topic	Item #	Checklist item	Location where item is reported
**TITLE**			
Title	1	Identify the report as a systematic review.	Page 1
**ABSTRACT**			
Abstract	2	See the PRISMA 2020 for Abstracts checklist.	Page 2
**INTRODUCTION**			
Rationale	3	Describe the rationale for the review in the context of existing knowledge.	Page 2
Objectives	4	Provide an explicit statement of the objective(s) or question(s) the review addresses.	Page 3
**METHODS**			
Eligibility criteria	5	Specify the inclusion and exclusion criteria for the review and how studies were grouped for the syntheses.	Page 4
Information sources	6	Specify all databases, registers, websites, organisations, reference lists and other sources searched or consulted to identify studies. Specify the date when each source was last searched or consulted.	Page 4
Search strategy	7	Present the full search strategies for all databases, registers and websites, including any filters and limits used.	Supplement
Selection process	8	Specify the methods used to decide whether a study met the inclusion criteria of the review, including how many reviewers screened each record and each report retrieved, whether they worked independently, and if applicable, details of automation tools used in the process.	Page 4 and 5
Data collection process	9	Specify the methods used to collect data from reports, including how many reviewers collected data from each report, whether they worked independently, any processes for obtaining or confirming data from study investigators, and if applicable, details of automation tools used in the process.	Page 5
Data items	10a	List and define all outcomes for which data were sought. Specify whether all results that were compatible with each outcome domain in each study were sought (e.g. for all measures, time points, analyses), and if not, the methods used to decide which results to collect.	Page 4
	10b	List and define all other variables for which data were sought (e.g. participant and intervention characteristics, funding sources). Describe any assumptions made about any missing or unclear information.	Page 4 and 5
Study risk of bias assessment	11	Specify the methods used to assess risk of bias in the included studies, including details of the tool(s) used, how many reviewers assessed each study and whether they worked independently, and if applicable, details of automation tools used in the process.	Page 6
Effect measures	12	Specify for each outcome the effect measure(s) (e.g. risk ratio, mean difference) used in the synthesis or presentation of results.	Page 6
Synthesis methods	13a	Describe the processes used to decide which studies were eligible for each synthesis (e.g. tabulating the study intervention characteristics and comparing against the planned groups for each synthesis (item #5)).	Page 6
	13b	Describe any methods required to prepare the data for presentation or synthesis, such as handling of missing summary statistics, or data conversions.	Page 6
	13c	Describe any methods used to tabulate or visually display results of individual studies and syntheses.	NA
	13d	Describe any methods used to synthesize results and provide a rationale for the choice(s). If meta-analysis was performed, describe the model(s), method(s) to identify the presence and extent of statistical heterogeneity, and software package(s) used.	Page 5
	13e	Describe any methods used to explore possible causes of heterogeneity among study results (e.g. subgroup analysis, meta-regression).	Page 5 and 6
	13f	Describe any sensitivity analyses conducted to assess robustness of the synthesized results.	NA
Reporting bias assessment	14	Describe any methods used to assess risk of bias due to missing results in a synthesis (arising from reporting biases).	NA
Certainty assessment	15	Describe any methods used to assess certainty (or confidence) in the body of evidence for an outcome.	NA

PRISMA 2020 Checklist

Section and Topic	Item #	Checklist item	Location where item is reported
**RESULTS**			
Study selection	16a	Describe the results of the search and selection process, from the number of records identified in the search to the number of studies included in the review, ideally using a flow diagram.	Page 7 and Figure 1
	16b	Cite studies that might appear to meet the inclusion criteria, but which were excluded, and explain why they were excluded.	Supplemental data 3
Study characteristics	17	Cite each included study and present its characteristics.	Page 7 - 8
Risk of bias in studies	18	Present assessments of risk of bias for each included study.	Page 8
Results of individual studies	19	For all outcomes, present, for each study: (a) summary statistics for each group (where appropriate) and (b) an effect estimate and its precision (e.g. confidence/credible interval), ideally using structured tables or plots.	Tables 1 to 3
Results of syntheses	20a	For each synthesis, briefly summarise the characteristics and risk of bias among contributing studies.	Page 9 -10
	20b	Present results of all statistical syntheses conducted. If meta-analysis was done, present for each the summary estimate and its precision (e.g. confidence/credible interval) and measures of statistical heterogeneity. If comparing groups, describe the direction of the effect.	Tables 1 to 3
	20c	Present results of all investigations of possible causes of heterogeneity among study results.	Page 9 -10
	20d	Present results of all sensitivity analyses conducted to assess the robustness of the synthesized results.	NA
Reporting biases	21	Present assessments of risk of bias due to missing results (arising from reporting biases) for each synthesis assessed.	NA
Certainty of evidence	22	Present assessments of certainty (or confidence) in the body of evidence for each outcome assessed.	NA
**DISCUSSION**			
Discussion	23a	Provide a general interpretation of the results in the context of other evidence.	Page 10-11
	23b	Discuss any limitations of the evidence included in the review.	Page 13
	23c	Discuss any limitations of the review processes used.	NA
	23d	Discuss implications of the results for practice, policy, and future research.	Page 14
**OTHER INFORMATION**			
Registration and protocol	24a	Provide registration information for the review, including register name and registration number, or state that the review was not registered.	Page 4
	24b	Indicate where the review protocol can be accessed, or state that a protocol was not prepared.	Page 4
	24c	Describe and explain any amendments to information provided at registration or in the protocol.	NA
Support	25	Describe sources of financial or non-financial support for the review, and the role of the funders or sponsors in the review.	Page 15
Competing interests	26	Declare any competing interests of review authors.	Page 15
Availability of data, code and other materials	27	Report which of the following are publicly available and where they can be found: template data collection forms; data extracted from included studies; data used for all analyses; analytic code; any other materials used in the review.	Page 15

From: Page MJ, McKenzie JE, Bossuyt PM, Boutron I, Hoffmann TC, Mulrow CD, et al. The PRISMA 2020 statement: an updated guideline for reporting systematic reviews. BMJ 2021;372:n71. doi: 10.1136/bmj.n71.
This work is licensed under CC BY 4.0. To view a copy of this license, visit https://creativecommons.org/licenses/by/4.0/

Supplemental data 2: Search Strategies

1. **Primary and updated search via databases**

Database	Search terms	Limiters	N (articles)
SPORTDiscus 2024-31-11	(TI "breast" OR AB "breast") AND (TI "cancer" OR AB "cancer" OR TI "carcinoma" OR AB "carcinoma" OR TI "tumor" OR AB "tumor" OR TI "tumour" OR AB "tumour") AND (TI "physical activity" OR AB "physical activity" OR TI "exercise" OR AB "exercise") AND (TI "randomized controlled trial" OR AB "randomized controlled trial" OR TI "RCT" OR AB "RCT") AND (DT 1989-2024)	Full Text; Peer Reviewed; English, Spanish or Portuguese	72
MEDLINE with Full Text 2024-31-11	("Breast"[Mesh] OR breast*[tiab]) AND ("Neoplasms"[Mesh] OR cancer*[tiab] OR carcinoma*[tiab] OR tumor*[tiab] OR tumour*[tiab]) AND ("Physical Activity"[Mesh] OR "Exercise"[Mesh] OR physical activit*[tiab] OR exercise*[tiab]) AND (Randomized Controlled Trial[Publication Type] OR randomized controlled trial*[tiab] OR RCT*[tiab]) AND ("1989/01/01"[Date - Publication] : "2024/12/31"[Date - Publication])	Full Text; Peer Reviewed; English, Spanish or Portuguese	1519
Google scholar Full Text 2024-31-11	"breast" AND ("cancer" OR "carcinoma" OR "tumor" OR "tumour") AND ("physical activity" OR "exercise") AND ("randomized controlled trial" OR "RCT") AND (1989..2024)	Full Text; Peer Reviewed; English, Spanish or Portuguese. First 200 results only, sort by relevance	1372
Lilacs Full Text 2024-31-11	(breast OR mama) AND (cancer OR carcinoma OR tumor OR tumour OR neoplasia) AND ("physical activity" OR exercise OR "atividade fisica") AND ("randomized controlled trial" OR RCT OR "ensaio clínico randomizado") AND (year_cluster:[1989 TO 2024])	Full Text; Peer Reviewed; English, Spanish or Portuguese	16
**Total**			**2979**

2. **Primary and updated search via other methods: 3 systematic reviews**

Title	N (Studies)

Tan TW, Tan HL, Chung YC. Effectiveness of resistance training in preventing sarcopenia among breast cancer patients undergoing chemotherapy: A systematic review and meta-analysis. Worldviews Evid Based Nurs. 2024 Dec;21(6):687-694. doi: 10.1111/wvn.12756. Epub 2024 Nov 21. PMID: 39572015.	11
Al-Mhanna SB, Batrakoulis A, Norhayati MN, Mohamed M, Drenowatz C, Irekeola AA, Afolabi HA, Gülü M, Alkhamees NH, Wan Ghazali WS. Combined Aerobic and Resistance Training Improves Body Composition, Alters Cardiometabolic Risk, and Ameliorates Cancer-Related Indicators in Breast Cancer Patients and Survivors with Overweight/Obesity: A Systematic Review and Meta-Analysis of Randomized Controlled Trials. J Sports Sci Med. 2024 Jun 1;23(2):366-395. doi: 10.52082/jssm.2024.366. PMID: 38841642; PMCID: PMC11149074.	17
Bao C, Feng Y, Huang J, Wang Z, Wang X, Hou Y, He C. The efficacy of exercise training for improving body composition in patients with breast cancer: A systematic review and meta-analysis of randomized controlled trials. Clin Rehabil. 2024 Sep;38(9):1188-1199. doi: 10.1177/02692155241232399. Epub 2024 May 24. PMID: 38794843.	14

Supplemental data 3: A list of excluded studies during full-text eligibility assessment (n = 91)

**Study Protocol (n = 4)**	1	Pilot trial testing the effects of exercise on chemotherapy-induced peripheral neurotoxicity (CIPN) and the interoceptive brain system
	2	Resistance training in advanced cancer: a phase II safety and feasibility trial-home versus hospital
	3	Optitrain: a randomised controlled exercise trial for women with breast cancer undergoing chemotherapy
	4	The women in steady exercise research (WISER) survivor trial: The innovative transdisciplinary design of a randomized controlled trial of exercise and weight-loss interventions among breast cancer survivors with lymphedema
**Not outcome available (n = 40)[a]**	5	Influence of cardiorespiratory training program on the intercellular adhesion molecule level in patients with postmastectomy syndrome
	6	Effects of physical exercise during adjuvant chemotherapy for breast cancer on long-term tested and perceived cognition: results of a pragmatic follow-up study
	7	Long Term Effects of a Social Capital-Based Exercise Adherence Intervention for Breast Cancer Survivors With Moderate Fatigue: A Randomized Controlled Trial
	8	Effects of an 18-Week Integrated Yoga Program on Cardiac Autonomic Function in Breast Cancer Patients Undergoing Adjuvant Chemotherapy: A Randomized Controlled Trial
	9	Effects of an educational physical activity intervention in young women with newly diagnosed breast cancer: Findings from the Young and Strong Study
	10	The effects of exercise and diet on oxidative stress and telomere length in breast cancer survivors
	11	Effects of exercise training on cardiac toxicity markers in women with breast cancer undergoing chemotherapy with anthracyclines: a randomized controlled trial
	12	The impact of mindfulness on cancer-related cognitive impairment in breast cancer survivors with cognitive complaints
	13	Improved Sleep Quality Is Associated with Reduced Insulin Resistance in Cancer Survivors Undertaking Circuit, Interval-Based Exercise
	14	The effect of exercise training on endothelial function in postmenopausal women with breast cancer under aromatase inhibitor therapy
	15	"Patterns of Fitbit Use and Activity Levels Among African American Breast Cancer Survivors During an eHealth Weight Loss Randomized Controlled Trial
	16	A randomized trial of exercise and diet on health-related quality of life in survivors of breast cancer with overweight or obesity
	17	Impact of resistance and aerobic exercise on sarcopenia and dynapenia in breast cancer patients receiving adjuvant chemotherapy: a multicenter randomized controlled trial
	18	Effect of exercise on the caloric intake of breast cancer patients undergoing treatment
	19	Effects of weight training on quality of life in recent breast cancer survivors: the Weight Training for Breast Cancer Survivors (WTBS) study
	20	Lower rate-pressure product during submaximal walking: a link to fatigue improvement following a physical activity intervention among breast cancer survivors
	21	Moderators of the effects of exercise training in breast cancer patients receiving chemotherapy: a randomized controlled trial

	22	Daughters and Mothers Against Breast Cancer (DAMES): main outcomes of a randomized controlled trial of weight loss in overweight mothers with breast cancer and their overweight daughters
	23	Effect of exercise training on C-reactive protein in postmenopausal breast cancer survivors: a randomized controlled trial
	24	Feasibility of an exercise and nutritional intervention for weight management during adjuvant treatment for localized breast cancer: the PASAPAS randomized controlled trial
	25	A pilot randomized controlled trial of a commercial diet and exercise weight loss program in minority breast cancer survivors
	26	The effect of aerobic exercise on metabolic and inflammatory markers in breast cancer survivors-a pilot study
	27	Effect of exercise on markers of inflammation in breast cancer survivors: the Yale exercise and survivorship study
	28	Randomised controlled trial of a home-based physical activity intervention in breast cancer survivors
	29	Impact of a mixed strength and endurance exercise intervention on insulin levels in breast cancer survivors
	30	Impact of a mixed strength and endurance exercise intervention on levels of adiponectin, high molecular weight adiponectin and leptin in breast cancer survivors
	31	Exercise training during chemotherapy preserves skeletal muscle fiber area, capillarization, and mitochondrial content in patients with breast cancer
	32	Effect of combination exercise training on metabolic syndrome parameters in postmenopausal women with breast cancer
	33	Effects of a weight loss intervention on body mass, fitness, and inflammatory biomarkers in overweight or obese breast cancer survivors
	34	Inflammation and psychosocial factors mediate exercise effects on sleep quality in breast cancer survivors: pilot randomized controlled trial
	35	Biobehavioral factors mediate exercise effects on fatigue in breast cancer survivors
	36	Effect of supervised and home exercise training on bone mineral density among breast cancer patients. A 12-month randomised controlled trial
	37	Effects of an exercise and hypocaloric healthy eating program on biomarkers associated with long-term prognosis after early-stage breast cancer: a randomized controlled trial
	38	A Randomized Controlled Trial of Exercise to Prevent Bone Loss in Premenopausal Women with Breast Cancer
	39	Evaluation of the effects of sensorimotor exercise on physical and psychological parameters in breast cancer patients undergoing neurotoxic chemotherapy
	40	Long-Term Physical Activity and Body Composition After Exercise and Educational Programs for Breast Cancer: A Randomized Controlled Trial From the Setouchi Breast Project-10
	41	Effect of Combining Impact-Aerobic and Strength Exercise, and Dietary Habits on Body Composition in Breast Cancer Survivors Treated with Aromatase Inhibitors
	42	Effects of aerobic exercise on neurocognitive function in postmenopausal women receiving endocrine therapy for breast cancer: The Exercise Program in Cancer and Cognition randomized controlled trial
	43	Supervised, structured and individualized exercise in metastatic breast cancer: a randomized controlled trial
	44	Effect of Multi-component Exercise Program on Body Composition and Physical, Emotional and Social well being in Breast Cancer Survivors
	45	Comparison of water- vs. land-based exercise for improving functional capacity and quality of life in patients living with and beyond breast cancer (the AQUA-FiT study): a randomized controlled trial

**Absence of inactive or less active control group (n = 12)[b]**	46	Healthy Moves to Improve Lifestyle Behaviors of Cancer Survivors and Their Spouses: Feasibility and Preliminary Results of Intervention Efficacy
	47	Effects of a combined aerobic and resistance exercise program in breast cancer survivors: a randomized controlled trial
	48	The effects of the mirror therapy on shoulder function in patients with breast cancer following surgery: a randomized controlled trial
	49	Comparison of Rehabilitation Training at Different Timepoints to Restore Shoulder Function in Patients With Breast Cancer After Lymph Node Dissection: A Randomized Controlled Trial"
	50	Manual Lymph Drainage With Progressive Arm Exercises for Axillary Web Syndrome After Breast Cancer Surgery: A Randomized Controlled Trial
	51	Effectiveness of Pilates-based exercises on upper extremity disorders related with breast cancer treatment
	52	Effect of combined Kinesiotaping and resistive exercise on muscle strength and quality of life in breast cancer survivors: a randomized clinical trial
	53	Implementation of a Mindful Walking Intervention in Breast Cancer Patients After Their Primary Oncologic Treatment: Results of a Qualitative Study Within a Randomized Controlled Trial
	54	EXERT-BC: A pilot study of an exercise regimen designed to improve functional mobility, body composition, and strength after the treatment for breast cancer
	55	Skeletal muscle mass, strength, and physical performance gains are similar between healthy postmenopausal women and postmenopausal breast cancer survivors after 12 weeks of resistance exercise training.
	56	Comparison of water- vs. land-based exercise for improving functional capacity and quality of life in patients living with and beyond breast cancer (the AQUA-FiT study): a randomized controlled trial.
**No Breast cancer data was available (n = 9)[c]**	57	Effects of exercise or metformin on myokine concentrations in patients with breast and colorectal cancer: A phase II multi-centre factorial randomized trial
	58	Can strength training or tai ji quan training reduce frailty in postmenopausal women treated with chemotherapy? A secondary data analysis of the GET FIT trial"
	59	Home-based prehabilitation with exercise to improve postoperative recovery for older adults with frailty having cancer surgery: the PREHAB randomised clinical trial
	60	A pilot randomized controlled trial using Baduanjin qigong to reverse frailty status among post-treatment older cancer survivors
	61	Dietary and Physical Activity Changes and Adherence to WCRF/AICR Cancer Prevention Recommendations following a Remotely Delivered Weight Loss Intervention for Female Breast Cancer Survivors: The Living Well after Breast Cancer Randomized Controlled Trial
	62	Hunger Training as a Self-regulation Strategy in a Comprehensive Weight Loss Program for Breast Cancer Prevention: A Randomized Feasibility Study"
	63	Effect of a Remotely Delivered Weight Loss Intervention in Early-Stage Breast Cancer: Randomized Controlled Trial",
	64	Effect of self-regulatory behaviour change techniques and predictors of physical activity maintenance in cancer survivors: a 12-month follow-up of the Phys-Can RCT
	65	Dose-dependent effects of aerobic exercise on clinically relevant biomarkers among healthy women at high genetic risk for breast cancer: A secondary analysis of a randomized controlled study

**No exercise intervention (n = 3)[d]**	66	Effect of the Lifestyle, Exercise, and Nutrition (LEAN) Study on Long-Term Weight Loss Maintenance in Women with Breast Cancer
	67	Randomized pilot test of a lifestyle physical activity intervention for breast cancer survivors
	68	Five-year follow-up of the OptiTrain trial on concurrent resistance and high-intensity interval training during chemotherapy for patients with breast cancer.
**Physiotherapy or oriental practices interventions (n = 8)[e]**	69	The efficacy of physiotherapy upon shoulder function following axillary dissection in breast cancer, a randomized controlled study
	70	A Randomized Trial on the Effect of Exercise Mode on Breast Cancer-Related Lymphedema
	71	The effects of yoga on shoulder and spinal actions for women with breast cancer-related lymphoedema of the arm: A randomised controlled pilot study
	72	Tai Chi Chuan for breast cancer survivors
	73	Short-term effects of a new resistance exercise approach on physical function during chemotherapy after radical breast cancer surgery: a randomized controlled trial
	74	Prospective surveillance and targeted physiotherapy for arm morbidity after breast cancer surgery: a pilot randomized controlled trial
	75	Effects of Nia exercise in women receiving radiation therapy for breast cancer
	76	The health effects of Baduanjin exercise (a type of Qigong exercise) in breast cancer survivors: A randomized, controlled, single-blinded trial
**No data available after the request (n = 7)[f]**	77	The effects of resistance exercise on appetite sensations, appetite related hormones and energy intake in hormone receptor-positive breast cancer survivors
	78	Effect of aerobic exercise on body weight and composition in patients with breast cancer on adjuvant chemotherapy
	79	A Pilot Randomized Controlled Trial on the Effects of a Progressive Exercise Program on the Range of Motion and Upper Extremity Grip Strength in Young Adults With Breast Cancer
	80	An exercise intervention for breast cancer survivors with bone loss
	81	The effect of exercise on body composition and bone mineral density in breast cancer survivors taking aromatase inhibitors
	82	Structured exercise improves physical functioning in women with stages I and II breast cancer: results of a randomized controlled trial
	83	The effect of exercise on body composition and bone mineral density in breast cancer survivors taking aromatase inhibitors
**Same data the other study that was ahead included (n = 3)[g]**	84	WISER Survivor Trial: Combined Effect of Exercise and Weight Loss Interventions on Insulin and Insulin Resistance in Breast Cancer Survivors
	85	WISER Survivor Trial: Combined Effect of Exercise and Weight Loss Interventions on Inflammation in Breast Cancer Survivors
	86	Effects of a 12-week supervised resistance training program, combined with home-based physical activity, on physical fitness and quality of life in female breast cancer survivors: the EFICAN randomized controlled
**Additional interventions alongside exercise (n = 5)[h].**	87	Short- and long-term impact of adapted physical activity and diet counseling during adjuvant breast cancer therapy: the "APAD1" randomized controlled trial
	88	Physical activity and health outcomes three months after completing a physical activity behavior change intervention: persistent and delayed effects

	89	Randomized Trial Comparing Telephone Versus In-Person Weight Loss Counseling on Body Composition and Circulating Biomarkers in Women Treated for Breast Cancer: The Lifestyle, Exercise, and Nutrition (LEAN) Study
	90	Stay on Track: A Pilot Randomized Control Trial on the Feasibility of a Diet and Exercise Intervention in Patients with Breast Cancer Receiving Radiotherapy
	91	Effect of Combining Impact-Aerobic and Strength Exercise, and Dietary Habits on Body Composition in Breast Cancer Survivors Treated with Aromatase Inhibitors

[a]Studies did not quantitatively or qualitatively report muscle strength, fat-free mass, functional exercise capacity and/or cardiorespiratory fitness outcomes defined in this review.

[b]Studies presented no control groups or control groups received any other exercise intervention.

[c]Studies presented data from breast cancer survivors mixed with other types of cancer, without specifying them.

[d]Studies were not intervention research or did not present an exercise intervention.

[e]Studies presented physiotherapy or oriental practices interventions such as Yoga, Tai-chi, Nia and others.

[f]Studies did not provide data after the request.

[g]Studies that used the same data as an included studied.

hStudies that involved additional interventions alongside exercise and whose outcomes could not be extracted separately

Supplemental data 4: Study characteristics.

Study	N	Control Group	Phase	Exercise mode	Delivery mode	Intervention duration	Frequency	Intensity	Adherence to exercise program	Outcomes measured
						(weeks)	(times/week)		(%)	
Ariza-Garcia et al. (18)	N = 39; C = 20 and EX = 19	UC with recommendations of PA	During treatment	Combined	Non-supervised	8	3	Low-to-moderate	73	HG strength, BIA lean mass, 6MWT
Battaglini et al. (19)	N = 20; C = 22 and EX = 21	UC	During treatment	Combined	Supervised	21	2	Low-to-moderate	100	1RM strength and Antropometric lean mass
Bertoli et al. (20)	N = 43; C = 22 and EX = 21	Relaxing and information program	Post-treatment	Resistance	Supervised	24	3	Low-to-moderate	NR	HG strength
Brown et al. (21)	N = 177, C = 90 and EX = 87	UC	Post-treatment	Combined	Supervised	52	3	Low-to-moderate	78	DXA lean mass
Bruno et al. (22)	N = 38; C = 20 and EX = 18	UC with recommendations of PA	Post-treatment	Aerobic	Supervised	12	2	Low-to-moderate	NR	BIA lean mass
Casla et al. (23)	N = 89; C = 44 and EX = 45	UC	Post-treatment	Combined	Supervised	12	2	Moderate-to-high	NR	BIA lean mass
Cešeiko et al. (24)	N = 55; C = 28 and EX = 27	UC	During treatment	Resistance	Supervised	12	2	Moderate-to-high	96	1RM strength
Cešeiko et al. (25)	N = 55; C = 28 and EX = 27	The control group was instructed to perform 3 sets of 10 chair rises twice a week for 12 weeks	During treatment	Resistance	Supervised	12	2	Moderate-to-high	96	1RM strength and 6MWT
Chang et al. (26)	N = 34; C = 17 and EX = 17	UC	Post-treatment	Combined	Supervised	12	3	Moderate-to-high	94	HG strength
Cornette et al. (27)	N = 42; C = 22 and EX = 20	UC	During treatment	Combined	Supervised	27	1	Moderate-to-high	88	1RM strength, 6MWT, direct VO2 test
Courneya et al. (28)	N = 164; C = 82 and EX = 82	UC	During treatment	Resistance	Supervised	17	3	Low-to-moderate	70	1RM strength, DXA lean mass, direct VO2 test

De Luca et al. (29)	N = 20; C = 10 and EX = 10	UC	Post-treatment	Combined	Supervised	24	2	Moderate-to-high	NR	1RM strength
Demark-Wahnefried et al. (30)	N = 58; C = 29 and EX = 29	Received information about diet	During treatment	Combined	Non-supervised	24	3	Moderate-to-high	81	DXA lean mass
Demark-Wahnefried et al. (31)	N = 32; C = 15 and EX = 17	Received basic nutritional counseling and upper-body progressive resistance training	Prehabilitation	Aerobic	Supervised	4	2	Low-to-moderate	NR	DXA lean mass, direct VO2 test
DeNysschen et al. (32)	N = 70; C = 34 and EX = 36	UC	During treatment	Aerobic	Non-supervised	48	3	Moderate-to-high	78	DXA lean mass
Diaz-Balboa et al. (33)	N = 122; C = 62 and EX = 60	usual care group received PA advice via telephone every two months with motivational interviewing by the physiotherapist until final assessment.	During treatment	Combined	Supervised	12	2	Moderate-to-high	NR	HG strength and direct VO2 test
Dieli-Conwright et al. (34)	N = 56; C = 27 and EX = 29	UC	Post-treatment	Combined	Supervised	16	3	Moderate-to-high	95	DXA lean mass
Dieli-Conwright et al. (35)	N = 100; C = 50 and EX = 50	UC	Post-treatment	Combined	Supervised	16	3	Moderate-to-high	95	DXA lean mass
Dieli-Conwright et al. (36)	N = 20; C = 20 and EX = 20	UC with na exercise program offered following the study period	Post-treatment	Combined	Supervised	16	2	Low-to-moderate	NR	DXA lean mass
Dobek et al. (37)	N = 44; C = 19 and EX = 25	Received whole body stretching and relaxation exercises	Post-treatment	Resistance	Supervised	52	3	Low-to-moderate	NR	1RM strength and DXA lean mass
Gnagnarella et al. (38)	N = 115; C = 58 and EX = 57	Received general information and recommendations	Post-treatment	Combined	Non-supervised	24	4	Low-to-moderate	NR	BIA lean mass

		for a healthy lifestyle								
Hagstrom et al. (39)	N = 23; C = 9 and EX = 14	UC	Post-treatment	Resistance	Supervised	16	3	Moderate-to-high	NR	Isometric strenth
Harvie et al. (40)	N = 24; C = 12 and EX = 12	UC	Post-treatment	Combined	Supervised	12	2	Moderate-to-high	NR	DXA lean mass
Herrero et al. (41)	N = 16; C = 8 and EX = 8	UC	Post-treatment	Combined	Supervised	8	3	Moderate-to-high	91	Resonance lean mass and direct VO2 test
Hiraoui et al. (42)	N = 32; C = 12 and EX = 20	UC	Post-treatment	Combined	Non-supervised	6	2	Moderate-to-high	100	6MWT
Huo t al. (43)	N = 111; C = 61 and EX = 50	Received a self-training manual comprising suggestions as well as exercise methods for arms and shoulders.	During treatment	Resistance	Supervised	1	4	Low-to-moderate	NR	HG strength
Husebø et al. (44)	N = 67; C = 34 and EX = 33	UC	Post-treatment	Combined	Non-supervised	17	3	Low-to-moderate	48	6MWT
Irwin et al. (45)	N = 75; C = 38 and EX = 37	UC	Post-treatment	Combined	Non-supervised	24	5	Moderate-to-high	82	DXA lean mass
Isanejad et al. (46)	N = 20; C = 10 and EX = 10	UC	Post-treatment	Aerobic	Supervised	12	3	Moderate-to-high	NR	Antropometric lean mass and direct VO2 test
Janelsins et al. (47)	N = 19; C = 10 and EX = 9	UC	Post-treatment	Aerobic	Supervised	12	3	Low-to-moderate	NR	BIA lean mass
Kim et al. (48)	N = 30; C = 15 and EX = 15	UC	Post-treatment	Resistance	Supervised	12	3	Moderate-to-high	NR	HG strength and 6MWT
Kim et al. (49)	N = 42; C = 19 and EX = 23	Received calcium and vitamin D supplementation	Post-treatment	Combined	Non-supervised	24	3	Low-to-moderate	69	HG strength
Lee and An, (50)	N = 24; C = 13 and EX = 11	UC	Post-treatment	Combined	Supervised	12	3	Moderate-to-high	NR	HG strength and BIA lean mass
Li et al. (51)	N = 40; C = 19 and EX = 21	Received health education program	During treatment	Aerobic	Supervised	12	3	Moderate-to-high	NR	HG strength, BIA lean mass and direct VO2 test
Matthews et al. (52)	N = 36; C = 14 and EX = 22	UC	Post-treatment	Aerobic	Non-supervised	12	3	Moderate-to-high	94	BIA lean mass

Mefferd et al. (53)	N = 76; C = 29 and EX = 47	UC wait-list control group	Post-treatment	Combined	Non-supervised	16	2	Moderate-to-high	80	DXA lean mass
Mijwell et al. (54)	N = 134; ; C = 60 and EX = 74	Received written information about physical activity recommendations.	During treatment	Resistance	Supervised	16	2	Moderate-to-high	83	HG strength and direct VO2 test
Min et al. (55)	N = 56; C = 28 and EX = 28	Received written information about general health and physical activity recommendations.	During treatment	Resistance	Non-supervised	24	4	Low-to-moderate	NR	Isometric strength and BIA lean mass
Mock et al. (56)	N = 43; C = 20 and EX = 23	UC	During treatment	Aerobic	Non-supervised	24	6	Moderate-to-high	50	12MWT
Møller et al. (57)	N = 256; C = 128 and EX = 128	Individual 12-week instructed home-based pedometer intervention and health counselling	During treatment	Combined	Supervised	12	2	Moderate-to-high	85	1RM strength, DXA lean mass, direct VO2 test
Murtezani et al. (58)	N = 62; C = 32 and EX = 30	UC	Post-treatment	Aerobic	Supervised	10	3	Moderate-to-high	84	12MWT
Musanti, (59)	N = 21; C = 12 and EX = 9	Received flexibility exercise	Post-treatment	Resistance	Non-supervised	12	3	Low-to-moderate	NR	6RM strength and indirect VO2 test
Naczk et al. (60)	N = 24; C = 12 and EX = 12	UC	Post-treatment	Resistance	Supervised	6	2	Moderate-to-high	NR	Isometric strength and BIA lean mass
Navarro-Sanz et al. (61)	N = 53; C = 24 and EX = 29	UC	Post-treatment	Combined	Supervised	12	2	Moderate-to-high	NR	HG strength, antropometric lean mass and indirect VO2 test
Nikander et al. (62)	N = 28; C = 14 and EX = 14	UC	During treatment	Combined	Non-supervised	12	3	Moderate-to-high	78	Isometric strength
Nikander et al. (63)	N = 77; C = 40 and EX = 37	UC	Post-treatment	Aerobic	Non-supervised	52	3	Moderate-to-high	NR	HG strength
Pagola et al. (64)	N = 23; C = 10 and EX = 13	Received moderate intensity exercise without supervision	Post-treatment	Combined	Supervised	16	2	Moderate-to-high	83	HG strength and DXA lean mass
Rogers et al. (65)	N = 41; C = 20 and EX = 21	Received written information about	Post-treatment	Aerobic	Non-supervised	12	3	Low-to-moderate	90	HG strength and indirect VO2 test

		physical activity recommendations.								
Rogers et al. (66)	N = 222; C = 112 and EX = 110	Received written information about physical activity recommendations.	Post-treatment	Aerobic	Non-supervised	12	3	Low-to-moderate	NR	Indirect VO2 test
Rogers et al. (67)	N = 222; C = 112 and EX = 110	Received written information about physical activity recommendations.	Post-treatment	Aerobic	Supervised	6	2	Low-to-moderate	98	Isometric strength
Roveda et al. (68)	N = 20; C = 10 and EX = 10	UC	Post-treatment	Aerobic	Non-supervised	12	2	Moderate-to-high	86	BIA lean mass and direct VO2 test
Santagnllo t al. (69)	N = 20; C = 9 and EX = 11	Received muscle stretching exercises twice a week	Post-treatment	Resistance	Supervised	12	3	Moderate-to-high	NR	1RM strength, DXA lean mass, indirect VO2 test
Santos et al. (70)	N = 25; C = 13 and EX = 12	UC	Post-treatment	Resistance	Supervised	8	1	Moderate-to-high	99	10RM strength and DXA lean mass
Schmidt et al. (71)	N = 49; C = 28 and EX = 21	nr	During treatment	Aerobic	Supervised	12	2	Moderate-to-high	NR	Isometric strength and BIA lean mass
Schmitz et al. (72)	N = 81; C = 41 and EX = 40	Received 13 weeks of exercise.	Post-treatment	Resistance	Supervised	48	2	Low-to-moderate	92	DXA lean mass
Schwartz et al. (73)	N = 45; C = 23 and EX = 22	UC	During treatment	Aerobic	Non-supervised	24	4	Moderate-to-high	NR	1RM and 12MWT
Sheppard et al. (74)	N = 31; C = 16 and EX = 15	UC	Post-treatment	Aerobic	Non-supervised	12	5	Low-to-moderate	70	Direct VO2 test
Swisher et al. (75)	N = 28; C = 10 and EX = 18	Received written information about physical activity recommendations.	Post-treatment	Aerobic	Supervised	12	3	Moderate-to-high	NR	Direct VO2 test
Winters-Stone et al. (76)	N = 48; C = 25 and EX = 23	Received muscle stretching and relaxation exercises	Post-treatment	Combined	Supervised	52	3	Low-to-moderate	64	1RM strength and DXA lean mass
Winters-Stone et al. (77)	N = 67; C = 31 and EX = 36	Received a low-intensity, non-weight-bearing stretching exercise	Post-treatment	Combined	Non-supervised	48	3	Low-to-moderate	57	DXA lean mass

Zhao et al. (78)	N = 60; C = 30 and EX = 30	Received written information about physical activity recommendations.	Post-treatment	Combined	Non-supervised	12	2	Low-to-moderate	NR	Antropometric lean mass
Reis et al. (79)	N = 26; C = 13 and EX = 13	UC	During treatment	Combined	Supervised	12	3	Moderate-to-high	NR	HG strength, antropometric lean mass and indirect VO2 test
Antunes et al. (80)	N = 91; C = 45 and EX = 43	UC	During treatment	Combined	Supervised	20	2	Moderate-to-high	73	HG strength
Garcia-Roca et al. (81)	N = 59; C = 28 and EX = 31	Received written information about physical activity recommendations.	During treatment	Combined	Supervised	24	2	Low-to-moderate	NR	HG strength, BIA lean mass, 6MWT
Vikmoen et al. (82)	N = 34; C = 17 and EX = 17	UC	During treatment	Resistance	Supervised	16	2	Moderate-to-high	72	1RM strength and plestimographylean mass
Casanovas-Álvarez et al. (83)	N = 64; C = 32 and EX = 32	UC	Prehabilitation	Combined	Supervised	16	2	Low-to-moderate	92	HG strength and 6MWT
Portela et al. (84)	N = 63; C = 35 and EX = 28	Received a low-intensity endurance exercise	Post-treatment	Aerobic	Non-supervised	24	5	Low-to-moderate	NR	6MWT
Esteban-Simón et al. (85)	N = 57; C = 28 and EX = 29	UC	Post-treatment	Resistance	Supervised	12	2	Low-to-moderate	NR	Isometric strength

BIA, bioelectrical impedance; DXA, Dual-energy X-ray Absorptiometry; NR, not reported; UC, usual care; RM, repetition maximum; MWT, minutes walking test.

Supplemental data 5: Risk of bias assessment of included studies using V.2 of the Cochrane risk of bias tool.

Study ID	Experimental	Comparator	Outcome	D1	D2	D3	D4	D5	Overall
Ariza-Garcia et al. (18)	Exercise	Control	Strength, fat-free mass and functional capacity	+	+	-	+	+	-
Battaglini et al. (19)	Exercise	Control	Strength and fat-free mass	!	+	+	!	+	!
Bertoli et al. (20)	Exercise	Control	Strength	!	+	-	+	!	-
Brown et al. (21)	Exercise	Control	Fat-free mass	-	+	+	+	+	-
Bruno et al. (22)	Exercise	Control	Fat-free mass	!	+	+	+	+	!
Casla et al. (23)	Exercise	Control	Fat-free mass	-	+	+	+	+	-
Cešeiko et al. (24)	Exercise	Control	Fat-free mass and cardiorespiratory fitness	!	+	+	!	+	!
Cešeiko et al. (25)	Exercise	Control	Strength nad functional capacity	!	+	+	!	+	!
Chang et al. (26)	Exercise	Control	Strength	!	+	+	+	+	!
Cornette et al. (27)	Exercise	Control	Strength, functional capacity and cardiorespiratory fitness	-	+	+	!	+	!
Courneya et al. (28)	Exercise	Control	Fat-free mass and cardiorespiratory fitness	!	+	+	!	+	!
De Luca et al. (29)	Exercise	Control	Strength	!	+	+	+	+	!
Demark-Wahnefried et al. (30)	Exercise	Control	Fat-free mass	!	+	+	+	+	!
Demark-Wahnefried et al. (31)	Exercise	Control	Fat-free mass and cardiorespiratory fitness	!	+	+	!	+	!
DeNysschen et al. (32)	Exercise	Control	Fat-free mass	!	+	+	+	+	!

+	Low risk
!	Some concerns
-	High risk

D1	Randomization process
D2	Deviations from the intended interventions
D3	Missing outcome data
D4	Measurement of the outcome
D5	Selection of the reported result

Diaz-Balboa et al. (33)	Exercise	Control	Strength and cardiorespiratory fitness	!	+	+	+	+	!
Dieli-Conwright et al. (34)	Exercise	Control	Fat-free mass	!	+	+	+	+	!
Dieli-Conwright et al. (35)	Exercise	Control	Fat-free mass	!	+	+	+	+	!
Dieli-Conwright et al. (36)	Exercise	Control	Fat-free mass	!	+	+	+	!	!
Dobek et al. (37)	Exercise	Control	Strength and fat-free mass	-	+	+	+	+	-
Gnagnarella et al. (38)	Exercise	Control	Fat-free mass	!	+	+	+	+	!
Hagstrom et al. (39)	Exercise	Control	Strength	!	+	+	+	+	!
Harvie et al. (40)	Exercise	Control	Fat-free mass	!	+	+	+	+	!
Herrero et al. (41)	Exercise	Control	Fat-free mass and cardiorespiratory fitness	-	+	+	+	+	-
Hiraoui et al. (42)	Exercise	Control	Functional capacity	-	+	+	+	+	-
Huo t al. (43)	Exercise	Control	Strength	-	+	+	+	+	-
Husebø et al. (44)	Exercise	Control	Functional capacity	!	+	+	+	+	!
Irwin et al. (45)	Exercise	Control	Fat-free mass	-	+	+	+	+	-
Isanejad et al. (46)	Exercise	Control	Fat-free mass and cardiorespiratory fitness	!	+	+	!	+	!
Janelsins et al. (47)	Exercise	Control	Fat-free mass	!	+	+	+	+	!
Kim et al. (48)	Exercise	Control	Strength and functional capacity	!	+	+	+	+	!
Kim et al. (49)	Exercise	Control	Strength	!	+	+	+	+	!
Lee and An, (50)	Exercise	Control	Strength and fat-free mass	!	+	+	+	+	!

Li et al. (51)	Exercise	Control	Strength, fat-free mass and cardiorespiratory fitness	-	+	+	+	+	-
Matthews et al. (52)	Exercise	Control	Fat-free mass	-	+	-	+	+	-
Mefferd et al. (53)	Exercise	Control	Fat-free mass	-	+	+	+	+	-
Mijwell et al. (54)	Exercise	Control	Strength and cardiorespiratory fitness	-	+	-	+	+	-
Min et al. (55)	Exercise	Control	Strength and fat-free mass	!	+	+	+	+	!
Mock et al. (56)	Exercise	Control	Functional capacity	!	+	+	+	+	!
Møller et al. (57)	Exercise	Control	Strength, fat-free mass and cardiorespiratory fitness	!	+	+	!	+	!
Murtezani et al. (58)	Exercise	Control	Functional capacity	-	+	+	+	+	-
Musanti, (59)	Exercise	Control	Strength and cardiorespiratory fitness	!	+	+	+	+	!
Naczk et al. (60)	Exercise	Control	Strength and fat-free mass	!	+	+	!	+	!
Navarro-Sanz et al. (61)	Exercise	Control	Strength, fat-free mass and cardiorespiratory fitness	-	+	+	+	+	-
Nikander et al. (62)	Exercise	Control	Strength	!	+	+	!	+	!
Nikander et al. (63)	Exercise	Control	Strength	-	+	+	+	+	-
Pagola et al. (64)	Exercise	Control	Strength and fat-free mass	!	+	+	+	+	!
Rogers et al. (65)	Exercise	Control	Strength and cardiorespiratory fitness	!	+	+	+	+	!
Rogers et al. (66)	Exercise	Control	Cardiorespiratory fitness	!	+	+	!	+	!
Rogers et al. (67)	Exercise	Control	Strength	!	+	+	!	+	!
Roveda et al. (68)	Exercise	Control	Fat-free mass and cardiorespiratory fitness	!	+	+	+	+	!

Santagnllo t al. (69)	Exercise	Control	Strength, fat-free mass and cardiorespiratory fitness	!	+	+	!	+	!
Santos et al. (70)	Exercise	Control	Strength and fat-free mass	!	+	+	!	+	!
Schmidt et al. (71)	Exercise	Control	Strength and fat-free mass	-	+	+	+	+	!
Schmitz et al. (72)	Exercise	Control	Fat-free mass	!	+	+	+	!	!
Schwartz et al. (73)	Exercise	Control	Strength and functional capacity	!	+	+	!	+	!
Sheppard et al. (74)	Exercise	Control	Cardiorespiratory fitness	!	+	+	+	+	!
Swisher et al. (75)	Exercise	Control	Cardiorespiratory fitness	-	+	+	+	+	-
Winters-Stone et al. (76)	Exercise	Control	Fat-free mass	-	+	+	+	!	!
Winters-Stone et al. (77)	Exercise	Control	Strength and fat-free mass	!	+	+	+	+	!
Zhao et al. (78)	Exercise	Control	Fat-free mass	!	+	+	!	+	!
Reis et al. (79)	Exercise	Control	Strength, fat-free mass and cardiorespiratory fitness	!	+	+	+	+	!
Antunes et al. (80)	Exercise	Control	Strength	!	+	+	+	+	!
Garcia-Roca et al. (81)	Exercise	Control	strength, fat-free mass, functional capacity	!	+	+	+	+	!
Vikmoen et al. (82)	Exercise	Control	Strength and fat-free mass	!	+	+	+	+	!
Casanovas-Álvarez et al. (83)	Exercise	Control	HG strength and functional capacity	!	+	+	+	+	!
Portela et al. (84)	Exercise	Control	Functional capacity	!	+	+	+	+	!
Esteban-Simón et al. (85)	Exercise	Control	Isometric strength	-	+	+	+	+	!

Supplemental data 6. Forest plot of subanalyses of each measurement

StrengthMeta Analysis

Group by Strength measure method	Study name	Std diff in means	Standard error	Variance	Lower limit	Upper limit	Z-Value	p-Value
Handgrip	Lee and An, (50)	-0,313	0,312	0,097	-0,924	0,298	-1,004	0,316
Handgrip	Pagola et al. (64)	0,000	0,421	0,177	-0,824	0,824	0,000	1,000
Handgrip	Ariza-Garcia et al. (18)	0,071	0,320	0,103	-0,557	0,699	0,221	0,825
Handgrip	Chang et al. (26)	0,107	0,343	0,118	-0,566	0,780	0,312	0,755
Handgrip	Kim et al. (49)	0,129	0,410	0,168	-0,674	0,933	0,316	0,752
Handgrip	Diaz-Balboa et al. (33)	0,167	0,181	0,033	-0,189	0,522	0,919	0,358
Handgrip	Kim et al. (48)	0,242	0,366	0,134	-0,476	0,960	0,660	0,509
Handgrip	Casanovas-Álvarez et al. (83)	0,320	0,252	0,063	-0,173	0,813	1,271	0,204
Handgrip	Nikander et al. (63)	0,345	0,230	0,053	-0,106	0,795	1,500	0,134
Handgrip	Navarro-Sanz et al. (61)	0,403	0,279	0,078	-0,143	0,950	1,448	0,148
Handgrip	Mijwell et al. (54)	0,507	0,176	0,031	0,161	0,853	2,873	0,004
Handgrip	Antunes et al. (80)	0,552	0,214	0,046	0,133	0,970	2,582	0,010
Handgrip	Huo t al. (43)	0,605	0,195	0,038	0,223	0,988	3,104	0,002
Handgrip	Rogers et al. (65)	0,672	0,321	0,103	0,042	1,301	2,092	0,036
Handgrip	Reis et al. (79)	0,819	0,408	0,167	0,018	1,619	2,005	0,045
Handgrip	Bertoli et al. (20)	1,333	0,337	0,114	0,672	1,994	3,952	0,000
Handgrip	Garcia-Roca et al. (81)	1,489	0,287	0,082	0,926	2,051	5,187	0,000
Handgrip	Li et al. (51)	6,632	0,806	0,650	5,052	8,213	8,226	0,000
Handgrip	Pooled	0,597	0,157	0,025	0,289	0,906	3,793	0,000
Handgrip	Prediction Interval	0,597			-0,688	1,882		
Isometric	Nikander et al. (62)	-0,381	0,381	0,145	-1,128	0,367	-0,998	0,318
Isometric	Rogers et al. (67)	0,145	0,134	0,018	-0,118	0,409	1,080	0,280
Isometric	Schmidt et al. (71)	0,280	0,290	0,084	-0,288	0,849	0,966	0,334
Isometric	Hagstrom et al. (39)	0,540	0,435	0,189	-0,312	1,391	1,241	0,214
Isometric	Esteban-Simón et al.(84)	0,647	0,272	0,074	0,115	1,180	2,382	0,017
Isometric	Min et al. (55)	1,610	0,308	0,095	1,007	2,213	5,235	0,000
Isometric	Naczk et al. (60)	1,736	0,479	0,229	0,798	2,675	3,625	0,000
Isometric	Pooled	0,618	0,256	0,066	0,116	1,121	2,413	0,016
Isometric	Prediction Interval	0,618			-1,039	2,276		
RM	Cornette et al. (27)	-0,591	0,316	0,100	-1,209	0,028	-1,872	0,061
RM	Vikmoen et al. (82)	0,346	0,346	0,119	-0,332	1,023	1,000	0,317
RM	Winters-Stone et al. (77)	0,421	0,292	0,085	-0,152	0,993	1,441	0,150
RM	Dobek et al. (37)	0,507	0,309	0,096	-0,098	1,113	1,641	0,101
RM	Møller et al. (57)	0,517	0,128	0,016	0,267	0,767	4,055	0,000
RM	Santos et al. (70)	0,773	0,415	0,172	-0,040	1,586	1,863	0,062
RM	Courneya et al. (28)	0,775	0,162	0,026	0,458	1,092	4,786	0,000
RM	Schwartz et al. (73)	0,789	0,310	0,096	0,182	1,396	2,549	0,011
RM	Musanti, (59)	0,831	0,459	0,211	-0,069	1,731	1,810	0,070
RM	Battaglini et al. (19)	1,093	0,479	0,230	0,153	2,033	2,280	0,023
RM	Cešeiko et al. (25)	1,223	0,294	0,086	0,647	1,799	4,162	0,000
RM	Cešeiko et al. (24)	1,570	0,308	0,095	0,965	2,175	5,090	0,000
RM	Santagnllo t al. (69)	1,611	0,517	0,267	0,599	2,624	3,119	0,002
RM	De Luca et al. (29)	1,829	0,533	0,284	0,785	2,873	3,435	0,001
RM	Pooled	0,768	0,143	0,020	0,488	1,047	5,386	0,000
RM	Prediction Interval	0,768			-0,184	1,720		
Overall	Pooled	0,680	0,098	0,010	0,489	0,872	6,964	0,000
Overall	Prediction Interval	0,680			-0,372	1,733		

Std diff in means and 95%CI

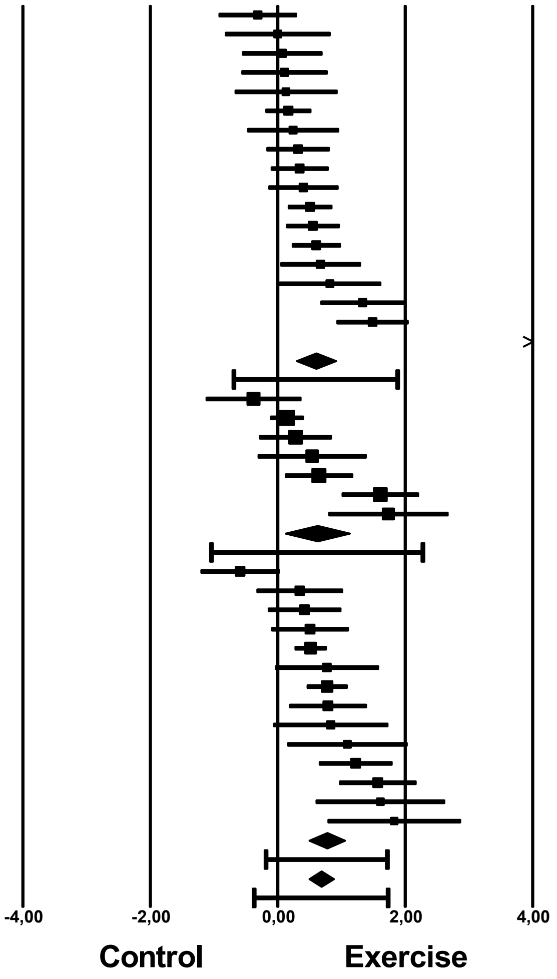

Random effects

Fat-Free Mass Meta Analysis

Group by<br>Fat-free mass measure method	Study name	Statistics for each study						
		Std diff in means	Standard error	Variance	Lower limit	Upper limit	Z-Value	p-Value
ANTROP	Reis et al. (79)	-0,569	0,400	0,160	-1,353	0,215	-1,422	0,155
ANTROP	Battaglini et al. (19)	0,039	0,447	0,200	-0,838	0,915	0,086	0,931
ANTROP	Navarro-Sanz et al. (61)	0,192	0,277	0,076	-0,350	0,734	0,695	0,487
ANTROP	Zhao et al. (78)	0,367	0,260	0,068	-0,143	0,877	1,410	0,158
ANTROP	Isanejad et al. (46)	0,731	0,462	0,213	-0,175	1,636	1,582	0,114
ANTROP	Pooled	0,168	0,184	0,034	-0,192	0,529	0,915	0,360
ANTROP	Prediction Interval	0,168			-0,736	1,073		
BIA	Min et al. (55)	-0,267	0,268	0,072	-0,794	0,259	-0,996	0,319
BIA	Li et al. (51)	-0,167	0,317	0,101	-0,788	0,455	-0,525	0,599
BIA	Naczk et al. (60)	-0,005	0,408	0,167	-0,805	0,795	-0,012	0,991
BIA	Ariza-Garcia et al. (18)	0,000	0,320	0,103	-0,628	0,628	0,000	1,000
BIA	Schmidt et al. (71)	0,004	0,289	0,083	-0,562	0,570	0,013	0,990
BIA	Lee and An, (50)	0,006	0,365	0,133	-0,710	0,721	0,015	0,988
BIA	Casla et al. (23)	0,011	0,212	0,045	-0,404	0,427	0,053	0,958
BIA	Matthews et al. (52)	0,013	0,342	0,117	-0,657	0,683	0,039	0,969
BIA	Janelsins et al. (47)	0,174	0,460	0,212	-0,728	1,076	0,378	0,706
BIA	Bruno et al. (22)	0,289	0,327	0,107	-0,351	0,929	0,885	0,376
BIA	Garcia-Roca et al. (81)	0,313	0,262	0,069	-0,202	0,827	1,191	0,233
BIA	Gnagnarella et al. (38)	0,350	0,188	0,035	-0,019	0,718	1,861	0,063
BIA	Roveda et al. (68)	0,588	0,323	0,105	-0,046	1,222	1,818	0,069
BIA	Pooled	0,119	0,080	0,006	-0,037	0,276	1,491	0,136
BIA	Prediction Interval							
DXA	Harvie et al. (40)	-0,573	0,417	0,174	-1,389	0,243	-1,376	0,169
DXA	DeNysschen et al. (32)	-0,062	0,239	0,057	-0,531	0,407	-0,258	0,796
DXA	Demark-Wahnefried et al. (31)	-0,056	0,354	0,126	-0,750	0,639	-0,158	0,875
DXA	Winters-Stone et al. (76)	-0,024	0,245	0,060	-0,505	0,456	-0,099	0,921
DXA	Mefferd et al. (53)	-0,005	0,236	0,056	-0,468	0,457	-0,023	0,981
DXA	Demark-Wahnefried et al. (30)	-0,005	0,263	0,069	-0,520	0,510	-0,020	0,984
DXA	Santos et al. (70)	-0,003	0,400	0,160	-0,787	0,782	-0,007	0,995
DXA	Dobek et al. (37)	0,000	0,304	0,093	-0,597	0,597	0,000	1,000
DXA	Møller et al. (57)	0,000	0,162	0,026	-0,317	0,317	0,002	0,999
DXA	Courneya et al. (28)	0,011	0,156	0,024	-0,295	0,318	0,073	0,941
DXA	Schmitz et al. (72)	0,021	0,222	0,049	-0,415	0,456	0,093	0,926
DXA	Winters-Stone et al. (77)	0,024	0,289	0,083	-0,542	0,590	0,083	0,934
DXA	Irwin et al. (45)	0,036	0,231	0,053	-0,417	0,489	0,155	0,877
DXA	Dieli-Conwright et al. (35)	0,154	0,448	0,201	-0,724	1,031	0,343	0,732
DXA	Pagola et al. (64)	0,182	0,421	0,178	-0,644	1,008	0,432	0,666
DXA	Brown et al. (21)	0,210	0,151	0,023	-0,085	0,506	1,396	0,163
DXA	Dieli-Conwright et al. (36)	0,430	0,271	0,073	-0,100	0,960	1,590	0,112
DXA	Santagnllo t al. (69)	0,432	0,455	0,207	-0,459	1,323	0,950	0,342
DXA	Dieli-Conwright et al. (34)	0,969	0,211	0,045	0,554	1,383	4,582	0,000
DXA	Pooled	0,111	0,068	0,005	-0,022	0,244	1,637	0,102
DXA	Prediction Interval	0,111			-0,231	0,453		
plestimografia	Vikmoen et al. (82)	0,035	0,310	0,096	-0,572	0,641	0,112	0,911
plestimografia	Pooled	0,035	0,310	0,096	-0,572	0,641	0,112	0,911
plestimografia	Prediction Interval							
Resonance	Herrero et al. (41)	-0,016	0,500	0,250	-0,996	0,964	-0,031	0,975
Resonance	Pooled	-0,016	0,500	0,250	-0,996	0,964	-0,031	0,975
Resonance	Prediction Interval							
Overall	Pooled	0,115	0,049	0,002	0,019	0,211	2,351	0,019
Overall	Prediction Interval	0,115			-0,002	0,232		

Std diff in means and 95% CI

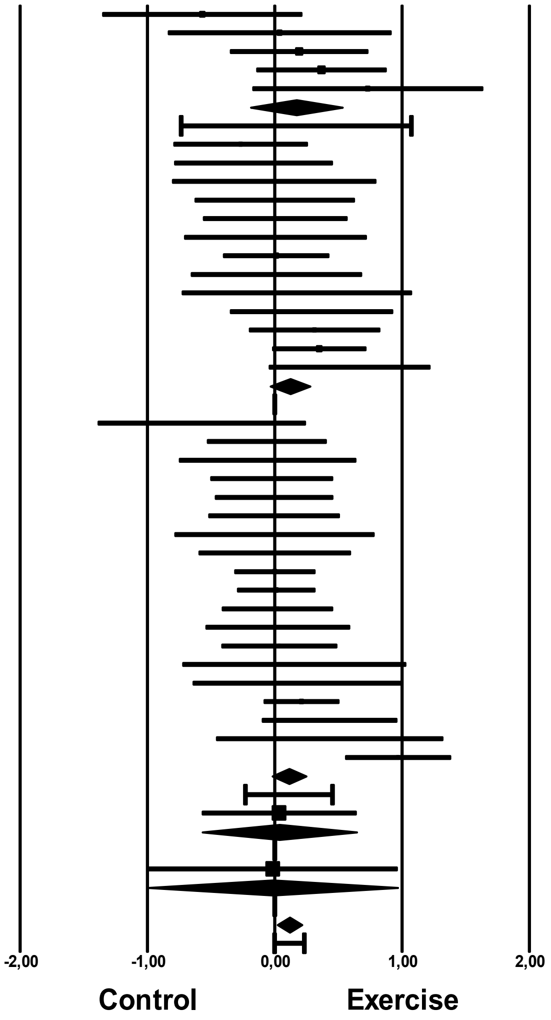

Random effects

Functional Exercise Capacity Meta Analysis

Group by Functional capacity mesasure method	Study name	Std diff in means	Standard error	Variance	Lower limit	Upper limit	Z-Value	p-Value
		Statistics for each study						
12MWT	Murtezani et al. (58)	0,594	0,260	0,067	0,085	1,103	2,287	0,022
12MWT	Mock et al. (56)	0,603	0,313	0,098	-0,009	1,216	1,931	0,054
12MWT	Schwartz et al. (73)	1,002	0,316	0,100	0,382	1,622	3,167	0,002
12MWT	Pooled	0,713	0,169	0,029	0,382	1,044	4,221	0,000
12MWT	Prediction Interval							
6MWT	Ariza-Garcia et al. (18)	0,235	0,321	0,103	-0,395	0,865	0,730	0,465
6MWT	Husebø et al. (44)	0,254	0,245	0,060	-0,227	0,734	1,034	0,301
6MWT	Portela et al. (84)	0,396	0,256	0,066	-0,105	0,898	1,548	0,122
6MWT	Cornette et al. (27)	0,507	0,314	0,099	-0,108	1,122	1,616	0,106
6MWT	Kim et al. (48)	0,548	0,316	0,100	-0,070	1,167	1,737	0,082
6MWT	Cešeiko et al. (25)	1,088	0,289	0,084	0,522	1,655	3,766	0,000
6MWT	Garcia-Roca et al. (81)	1,333	0,288	0,083	0,768	1,897	4,625	0,000
6MWT	Hiraoui et al. (42)	2,984	0,522	0,272	1,961	4,007	5,717	0,000
6MWT	Casanovas-Álvarez et al. (83)	3,664	0,409	0,167	2,862	4,466	8,956	0,000
6MWT	Pooled	1,169	0,333	0,111	0,515	1,822	3,505	0,000
6MWT	Prediction Interval	1,169			-1,196	3,533		
Overall	Pooled	0,806	0,151	0,023	0,511	1,101	5,349	0,000
Overall	Prediction Interval	0,806			-0,977	2,589		

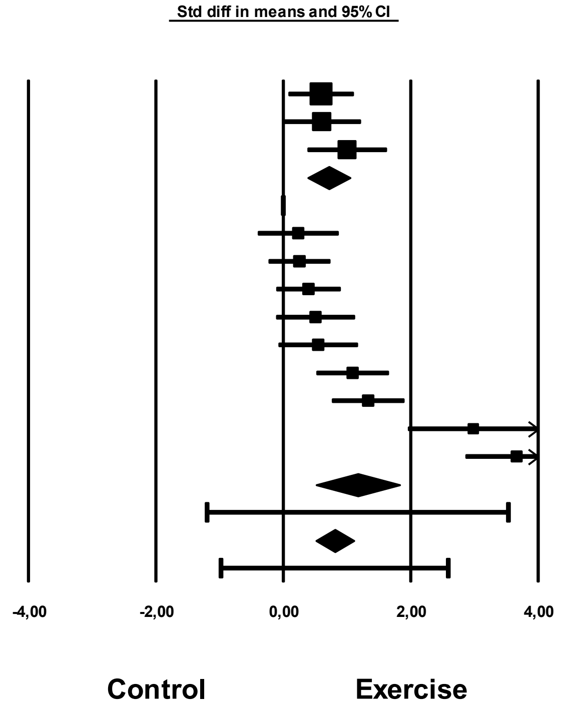

Random effects

Cardiorespiratory Fitness Meta Analysis

Group by VO2 measure method	Study name	Std diff in means	Standard error	Variance	Lower limit	Upper limit	Z-Value	p-Value
		Statistics for each study						
direct VO2peak	Swisher et al. (75)	-0,304	0,396	0,157	-1,081	0,473	-0,768	0,443
direct VO2peak	Herrero et al. (41)	0,049	0,500	0,250	-0,932	1,029	0,097	0,923
direct VO2peak	Demark-Wahnefried et al. (31)	0,095	0,354	0,126	-0,600	0,790	0,268	0,788
direct VO2peak	Roveda et al. (68)	0,113	0,448	0,200	-0,764	0,990	0,252	0,801
direct VO2peak	Courneya et al. (28)	0,122	0,156	0,024	-0,185	0,428	0,777	0,437
direct VO2peak	Sheppard et al. (74)	0,197	0,360	0,130	-0,509	0,903	0,547	0,584
direct VO2peak	Møller et al. (57)	0,399	0,126	0,016	0,152	0,647	3,161	0,002
direct VO2peak	Cornette et al. (27)	0,464	0,313	0,098	-0,149	1,078	1,483	0,138
direct VO2peak	Diaz-Balboa et al. (33)	0,467	0,184	0,034	0,107	0,826	2,543	0,011
direct VO2peak	Isanejad et al. (46)	1,271	0,490	0,240	0,310	2,232	2,593	0,010
direct VO2peak	Li et al. (51)	4,588	0,603	0,363	3,407	5,770	7,611	0,000
direct VO2peak	Mijwell et al. (54)	6,104	0,411	0,169	5,298	6,911	14,839	0,000
direct VO2peak	Pooled	1,082	0,378	0,143	0,341	1,823	2,861	0,004
direct VO2peak	Prediction Interval	1,082			-1,835	3,998		
Indirect VO2max	Navarro-Sanz et al. (61)	-0,041	0,276	0,076	-0,582	0,500	-0,149	0,882
Indirect VO2max	Rogers et al. (66)	0,178	0,135	0,018	-0,086	0,442	1,323	0,186
Indirect VO2max	Musanti, (59)	0,275	0,443	0,196	-0,593	1,143	0,621	0,535
Indirect VO2max	Rogers et al. (65)	0,299	0,314	0,099	-0,317	0,914	0,951	0,342
Indirect VO2max	Reis et al. (79)	2,066	0,486	0,236	1,114	3,018	4,254	0,000
Indirect VO2max	Santagnello t al. (69)	2,129	0,562	0,315	1,028	3,230	3,791	0,000
Indirect VO2max	Pooled	0,682	0,297	0,088	0,100	1,265	2,295	0,022
Indirect VO2max	Prediction Interval	0,682			-1,243	2,608		
Overall	Pooled	0,835	0,234	0,055	0,377	1,293	3,572	0,000
Overall	Prediction Interval	0,835			-1,420	3,090		

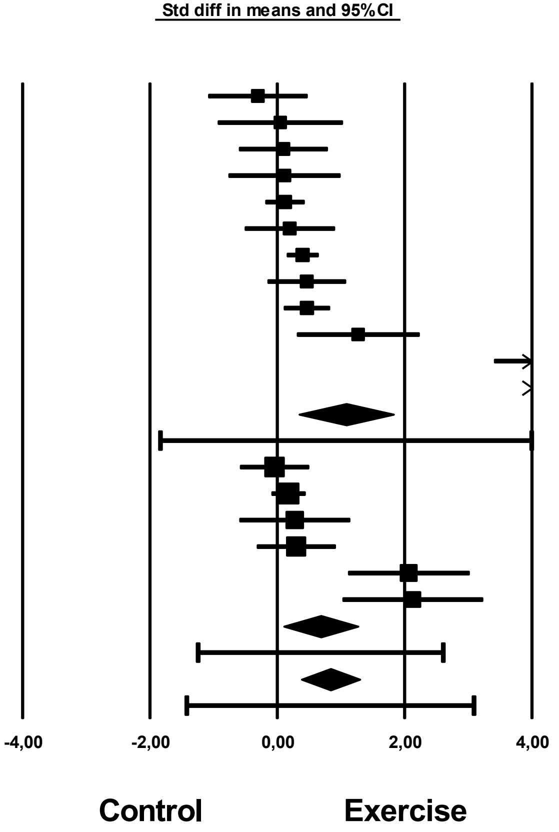

Random effects
